# Abstracts from the International Congress of the Society for Microbial Ecology and Disease SOMED 2013 Proceedings September 23th–26th 2013, Kosice, Slovakia

**DOI:** 10.3402/mehd.v24i0.23078

**Published:** 2013-12-13

## ORAL, September 23th–26th

## Abstracts of Oral Presentations

### Phage-Mediated Bioprocessing in Decontamination of Dairy Products

#### **Aleshkin, V.A.**^1^, Belousov, D.^2^, Aleshkin, A.^1^, Volozhantsev, N.^3^, Svetoch, E.^3^, Afanas'ev, S.^1^, Kiseleva, I.^1^, Taraseeva, O.^1^

^1^Gabrichevsky Moscow Research Institute of Epidemiology and Microbiology, Moscow, Russian Federation; ^2^Phage Technology s.r.o., Bratislava, Slovakia; ^3^State Research Center for Applied Microbiology & Biotechnology, Obolensk, Russian Federation

*Introduction:* The use of chemical decontaminants in the food industry impairs the quality and ecological purity of products, as well as increasing the risk that antibiotic-resistant strains of food pathogens will emerge. The development of a new decontamination method – bioprocessing based on bacteriophages – will reduce the risks of sporadic cases and outbreaks of food-borne infections, and also help retain the nutritional value and palatability of food products. This study aims to develop a method for phage-mediated decontamination of milk experimentally infected with *Staphylococcus aureus* and *Escherichia coli* strains. *Methods:* Using our pool of reference strains and strains isolated from patients as indicators of bacterial cultures, we isolated from the environment production-relevant *S. aureus* (StaK) and *E. coli* (EcD4) bacteriophages. Using microbiological, biochemical, and molecular genetics methods, their biological properties were studied and their safety for laboratory animals and humans was confirmed. Preliminarily boiled milk (3 min at 100°C, 10 ml each sample) was contaminated with 1 ml of 18 h cultures of *S. aureus* 2075 and *E. coli* K12 C600 strains at 10^7^ CFU/ml. Then 15 min after the contamination, the experimental samples were decontaminated with bacteriophages StaK and EcD4 in various titers (10^7^, 10^8^, and 10^9^ PFU/ml). Control samples were treated with 1 ml of physiological solution instead of the bacteriophages. The number of bacterial cells in both control and experimental samples (stored at 4±2°C) was estimated before the bacteriophage was introduced, and additionally 2, 4, 6, and 24 h after phage decontamination. *Results:* The concentration of *S. aureus* in the milk was 10^6^ CFU/ml 15 min after the contamination of both control and experimental samples. However, 2 h after StaK was introduced into the experimental samples, 99.955% of *S. aureus* was eliminated compared to the control samples when the StaK bacteriophage was used at the titer of 10^7^ PFU/ml, 99.984% at 10^8^ PFU/ml, and 99.985% at 10^9^ PFU/ml. Afterwards, the effect of decontamination increased, reaching over 99.999% after 24 h in all of the experimental samples: the *S. aureus* level in control samples was 10^9^ CFU/ml, whereas in the experimental group it was 3.2×10^3^, 3.6×10^2^, and 2.0×10^1^ CFU/ml when StaK was used in titers of 10^7^, 10^8^, and 10^9^ PFU/ml, respectively. The content of *E. coli* in all of the milk samples 15 min after contamination was also 10^6^ CFU/ml. After 2 h, elimination in experimental samples was 99.97% when the EcD4 phage was used in the titer of 10^7^ PFU/ml and up to 99.99% at 10^9^ PFU/ml. *E. coli* was completely lysed in 10^8^ and 10^9^ PFU/ml EcD4-treated samples, and was 4.0×10^2^ CFU/ml when the phage was used in the titer of 10^7^ PFU/ml. Meanwhile, the level of *E. coli* in the control samples continuously increased (from 10^7^ after 4 h to 10^9^ CFU/ml after 24 h). *Discussion:* This study demonstrates the StaK and EcD4 bacteriophages have high specific lytic activity against bacterial strains able to contaminate milk during its harvest and processing, and cause food-borne infections. The use of a new method of milk decontamination – phage-mediated bioprocessing – completely retains the nutritional value and palatability of the product.

Keywords: *S. aureus*; *E. coli*; phage-mediated bioprocessing; milk

### The Consequences of Different Antibiotic Treatments for Antimicrobial Resistance Genes Present in Gram-Negative Bacteria Isolated from Volunteers

#### **Anjum, M.F**., Kirchner, M., Card, R., Mafura, M., Hunt, T. Animal Health and Veterinary Laboratories Agency, UK

*Introduction:* In this study, a total of 3694 Gram-negative bacteria, mainly isolated from the feces and saliva, were gathered over 1 year to determine the effect of four different antibiotic treatments (minocycline, amoxicillin, ciprofloxacin, clindamycin) on the composition of antibiotic resistance genes present in these bacteria in human volunteers. *Methods:* An expanded Gram-negative microarray was used for detecting antibiotic resistance genes in aerobic and anaerobic Gram-negative bacteria isolated from a range of samples from different treatment and placebo groups. The largest proportion of isolates received from all treatment and placebo groups were from feces and were *Escherichia coli*. *Results*: Using our microarray, the diversity of resistance genes in aerobic bacteria was much greater for aerobic bacteria than for anaerobic bacteria irrespective of treatment; up to 50 different genes were detected in aerobic isolates and only up to eight different resistance genes in anaerobes. Also, *E. coli* generally harbored a much greater number of resistances than non-*E. coli* isolates, with the maximum number being 15. The array results also indicated that certain resistance genes were much more prevalent than others, with *bla*_TEM_ being the most prevalent across all groups, irrespective of treatment. This suggests widespread circulation of mobile genetic elements carrying this resistance gene. Anaerobic bacteria were unlikely to harbor resistance genes common in aerobes, although *sul2* was also detected in a small number of anaerobic bacteria. The data showed that the percentage of isolates harboring resistances to three or more classes of antibiotic were similar for all treatment groups including the placebos, at approximately 36%. Further analysis indicated that in the amoxicillin treatment groups, the percentage of multi-resistant isolates increased at day 11 post-treatment. We also noted a dramatic decrease in the percentage of isolates with no resistance genes in the ciprofloxacin and clindamycin groups following treatment, indicating a possible effect of antibiotic treatment on the resident microbiota. In some participants, conserved resistance gene sets were identified across multiple time-points for all treatment groups, including placebo. For selected examples, pulse field gel electrophoresis (PFGE) was performed and showed that isolates with the same resistance genes generally had identical PFGE profiles, even when collected from visits that were months apart. *Discussion:* These results indicate multi-drug resistant *E. coli* to be currently more prevalent in the individuals studied than other Gram-negative aerobic bacteria. We also noted the persistence of some isolates harboring resistance genes to multiple antibiotic classes, which was maintained in the absence of any antibiotic treatment. We did not detect any long-term changes to the overall antibiotic resistance gene composition from cultivable Gram-negative bacteria as a consequence of treatment with these antibiotics.

Keywords: microarray; antibiotic resistance; *Escherichia coli*

### Sampling the Human Microbiome for Antibiotic Resistance Genes Using Two Metagenomic Approaches

#### **Anjum, M.F**.^2^, Card, R.^1^, Warburton, P.^3^, MacLaren, N.^1^, Mullany, P.^2^, Allan, E.^2^
^1^Animal Health and Veterinary Laboratories Agency, UK; ^2^University College London, London, UK; ^3^Anglia Ruskin University, Cambridge, UK

*Introduction:* Antibiotic treatment can have profound impacts on the microbial population diversity, as has been shown using metagenomic approaches such as 16S rRNA 454 pyrosequencing. However, these methods do not provide insight into the associated antimicrobial resistance (AMR) genes that may be present. The aim of this study was to investigate the presence of AMR genes within the oral and fecal microbiota of healthy adult human volunteers. *Methods:* Two metagenomic approaches were employed: a DNA–DNA hybridization-based method and a function-based screen. The DNA–DNA hybridization-based method employed a microarray capable of detecting over 70 clinically relevant AMR genes. In the function-based approach, oral metagenomic DNA was cloned into an heterologous host (*Escherichia coli*) and screened for the expression of resistance to ampicillin or sulphonamide. Resistant clones were sequenced, annotated, and the presence of AMR genes deduced based on sequence homology. In order to place the detected AMR genes within the context of the microbiota, the microbial profiles of the samples were determined using 454 pyrosequencing of 16S rRNA gene amplicons. *Results:* AMR genes were detected by microarray in every sample. The most commonly detected genes were *ermB*, *vatE*, *bla*_TEM_, and *sul2*, all of which often reside on mobile genetic elements such as plasmids and integrons. In the ampicillin resistance function-based screen, all clones recovered were from *Haemophilus parainfluenzae* and possessed genes encoding a predicted AcrAB efflux pump. For the sulphonamide resistance screen, the clones recovered were from three species: *Neisseria subflava*, *Veillonella parvula*, and *Streptococcus infantis*. All clones had a *folP* gene encoding mutant dihydropteroate synthase, the target of sulphonamide action. The microbial profiles obtained were in general agreement with those reported in other studies of the healthy human oral and fecal microbiomes, and showed that the relative abundance of bacterial genera was similar between the different samples. *Discussion:* This study revealed the presence of genes that confer resistance to several classes of antibiotics in the microbiome of healthy human volunteers. The antibiotic resistance genes detected by functional screening were all recovered from the chromosomes of commensal species naturally resident in the human oral microbiota. These species are opportunistically pathogenic and capable of exchanging DNA with related pathogenic species. Genes identified by microarray were not recovered in the activity-based screen, indicating that these two methods are complementary in facilitating the identification of a range of resistance mechanisms. The results obtained show that the microbiota of healthy humans can serve as a reservoir for (potentially) transferable antimicrobial resistance genes. In future, the methods described in this study could be used to monitor changes in the resistome in response to antibiotic therapy.

Keywords: metagenome; antibiotic resistance genes; pathogens

### The Role of Transferable Plasmids in the Resistance and Fitness of Gram-negative Bacteria

#### **Anjum, M.F.**^1^, AbuOun, M.^1^, Thomas, Ch.^2^, Kirchner, M.^1^, Mafura, M.^1^
^1^Animal Health and Veterinary Laboratories Agency, UK; ^2^University of Birmingham, UK

*Introduction:* Plasmids are extrachromosomal double-stranded circular DNA elements that can harbor many different types of genes offering advantage to the host such as conferring resistance or toxin production. To study the importance of plasmids to their natural host, a plasmid-free derivative can be made using the pCURE system that targets IncF replicon plasmids. Four strains isolated during a longitudinal study on human volunteers treated with antibiotics (minocycline or amoxicillin) or placebo, were used to assess the contribution of the IncF plasmids on their native *Escherichia coli* host. *Methods:* Four kanamycin-sensitive *E. coli* isolates from the aforementioned groups were chosen for pCURE2 plasmid curing analysis. First the isolates were screened for the presence of native plasmids by plasmid profiling, the presence of plasmid incompatibility groups by PCR-based replicon typing (PBRT), and typed by *E. coli* multi-locus sequence type (MLST). Plasmid curing was achieved by either transformation or conjugation of the pCURE2 plasmid into each isolate with selection on kanamycin. Following curing of the native IncF plasmid, pCURE2-free segregants were selected by growth in the presence of 5% sucrose, and confirmed by plasmid profiling and PBRT. The replicon types, addiction system, antibiotic resistances, and effect on growth/respiration of a diverse range of metabolic effectors and stress inducers (phenotype microarrays, PM) were used to compare the wild-type and plasmid-cured derivatives. *Results:* The four isolates belonged to MLST ST10 and ST95 (types predominantly found in humans). Each isolate harbored between two and four plasmids of various sizes. Curing of the IncF plasmid resulted in removal of a 150 kb plasmid in all isolates. Molecular comparison of wild-type isolates and the cured derivatives revealed loss of various antibiotic resistance genes and addiction systems in three of the four isolates, while no change was observed in one isolate. PM analysis identified additional phenotypic fitness characteristics attributed to IncF plasmids in each isolate including antibiotic sensitivity. In a few instances, curing of the IncF plasmid resulted in increased fitness compared to wild-type when grown in the presence of an effector or stress inducer (e.g. colistin or potassium tellurite). *Discussion:* IncF plasmids harbored by human fecal *E. coli* were successfully cured from their native host using a directed stress-free approach (pCURE2). Each isolate harbored a plasmid of approximately 150 kb which carried different addiction systems and antimicrobial resistance genes, conferring differing phenotypes. In the majority of cases, the cured plasmid offered a fitness advantage to the host when exposed to a range of compounds. Further studies are under way to determine the molecular basis for the phenotypes observed.

Keywords: plasmids; fitness; antibiotics resistance

### The Dynamics of Fecal Microbiota During Selective Digestive Decontamination in Intensive Care Unit Patients

#### **Bello Gonzalez, T. de J**., Van Passel, M.W.J, Smidt, H. Wageningen University, The Netherlands

*Introduction:* Patients in intensive care units (ICUs) are at risk for infections caused by antibiotic-resistant pathogens, and such infections are associated with increased morbidity, mortality, and health care costs. Recent studies have shown that prophylactic antibiotic therapy improves patient survival by preventing infections caused by opportunistic pathogens originating from the patient's own microbiota. However, in these studies intestinal colonization was only assessed by conventional microbiological cultures of rectal swabs during selective digestive decontamination (SDD). It cannot be excluded that resistant bacteria are present in the bowel in low quantities but that the administered antibiotics suppress their growth leading to negative cultures. In this case, rapid growth of resistant bacteria could occur in patients after discontinuation of SDD. To this end, cultivation-independent fingerprinting can more comprehensively reveal changes in the gut microbiota associated with the differential presence of antibiotic resistance genes. The aim of this study is determine the within-host dynamics of the ICU patient microbiota before, during, and after SDD by phylogenetic profiling of fecal samples using the human intestinal tract chip (HIT Chip). *Methods:* To determine the effect of SDD on the phylogenetic profile and functional repertoire of the gut microbiota of ICU patients, we collected fecal samples from seven patients hospitalized in the ICU of Utrecht Medical Centre in 2011–2012. At three time points, samples were collected from each patient (day 0, 7 days and 14 days after discharge). Total DNA was extracted using a standardized protocol and the phylogenetic profiles of the gut microbiota were determined by HIT Chip. The HIT Chip is a phylogenetic array with over 4,800 tiling oligonucleotides targeting the V1 or the V6 region of the 16S rRNA gene from 1,132 microbial phylotypes present in the human gastrointestinal tract. *Results:* Phylogenetic profiling of fecal samples revealed the dynamic nature of the human gut microbiota during hospitalization in the ICU. The Bacteroidetes and Firmicutes (*Clostridium* cluster IV and XIVa) represented the majority of the gut microbiota during hospitalization. Their relative abundance changed considerably during hospitalization. During and after hospitalization, the bacilli group was significantly increased and Proteobacteria were suppressed. *Conclusions:* SDD therapy has a profound effect on the composition of the gut microbiota in ICU patients. A significant difference in the composition of the microbiota was observed between patients. However, the group of Proteobacteria found during the SDD therapy was significantly reduced compared with the bacilli group, which was found to be increased during the therapy. Our results indicated that the selective pressure on the gut microbiota is effective against overgrowth of potentially pathogenic aerobic bacteria and yeast but the effect on anaerobic bacteria including *Bacteroides* spp. and the *Clostridium* cluster group should also be considered.

Keywords: SDD; gut microbiota; microarray

### Characterization of Antibiotic Resistance Genes Using a Metagenomic Library from a Human Gut Microbiota Enrichment Culture

#### **Bello Gonzalez, T. de J.**^1^, Bülow, E.^2^, Van Schaik, W.^2^, Van Passel, M.W.J^1^, Smidt, H.^1^
^1^Wageningen University, The Netherlands; ^2^University Medical Center Utrecht, Utrecht, The Netherlands

*Introduction:* The human gut microbiota comprises 10^13^–10^14^ bacterial cells, and plays an important role in maintaining intestinal homeostasis. When the microbiota is perturbed (e.g. during the administration of antibiotics), the number of microorganisms is reduced, and resistance to colonization by potential pathogens is decreased. This could lead to overgrowth by naturally resistant microorganisms, or the establishment of new resistant pathogenic bacteria. The administration of prophylactic antibiotic therapy in the intensive care unit (ICU) contributes to the eradication of potential pathogenic bacteria while presumably leaving the commensal microbiota undisturbed. The aim of this study was to determine the presence of antibiotic resistance genes in the fecal microbiota of a patient who received antibiotic prophylaxis (selective oropharyngeal tract decontamination, SOD) during ICU hospitalization. *Methods:* The composition of the fecal microbiota of this patient was determined using the human intestinal tract chip (HIT Chip). A fosmid library (2.4 Gbp in size, insert size 40 Kb) was constructed using the pCC1FOSTM vector from an anaerobic liquid enrichment culture (PYG medium with tetracycline, erythromycin, and ampicillin) of a fecal sample collected from the patient who received a daily oral application of 0.5 g of a paste containing 2% polymyxin, 2% tobramycin, and 2% amphotericin B. The fosmid library was screened in *Escherichia coli* (EPI300) for antibiotic resistant clones to identify the antibiotic resistance determinants. *Results:* Gut microbiota compositional analysis using the HIT Chip revealed the presence of Bacteroidetes (53%), Firmicutes (40%, mostly *Clostridium* clusters XIVa and IV), and Actinobacteria (6%) as dominant phyla. The screening of the fosmid library revealed the presence of multiple clones resistant to ampicillin (100 µg/ml), tobramycin (25 µg/ml), erythromycin (500 µg/ml), or tetracycline (10 µg/ml). No clones resistant to imipenem were found. The identification of the putative bacterial source of the cloned insert sequence showed the presence of *Blautia* spp. (erytromycin and tetracycline resistance), *Enterobacter* spp., and *Escherichia coli* (ampicillin and tetracycline resistance) and *Bacteroides* spp. (erythromycin, tetracycline, and ampicillin resistance). Fosmid insert sequence analysis identified in *E. coli* a β-lactamase gene CTX-M_15_ which conferred resistance to ampicillin. *Conclusions:* We showed the presence of antibiotic resistant strains (*Enterobacter* spp., *Escherichia coli*, *Blautia* spp., and *Bacteroides* spp.) in a patient receiving prophylactic antibiotic treatment during an ICU stay, suggesting a range of resistance genes. The presence of antibiotic resistance genes in the gut microbiota may enhance the possibility of resistance gene transfer from gut commensals to opportunistic pathogens.

Keywords: antibiotic resistance genes; metagenomic library; human gut microbiota

### When and How Long to Administer Probiotic Bacteria in Gastrointestinal Diseases

#### Bertazzoni Minelli, E. University of Verona, Italy

*Introduction:* Due to the high heterogenicity of studies, there is no definitive evidence as to whether probiotics are helpful. Scientific substantiation of health claims requires adequate well-designed studies. The selection of strains, the dose, the modality of administration, and also the timing and duration of treatment are important points that need to be carefully evaluated for various gastrointestinal (GI) conditions. *Discussion:* The treatment approaches are different in acute and chronic pathology. Meta-analyses suggested that various probiotics significantly reduced infectious diarrhea (viral, bacterial) and antibiotic-associated diarrhea. The early administration of probiotics was beneficial for the treatment of infectious diarrhea, while later administration was not. The early administration of *Lactobacillus rhamnosus* GG for the treatment of acute rotavirus diarrhea (5×10^9^ CFU) reduced the symptoms better than the delayed administration of a higher dose (1×10^11^CFU). Early administration in preterm low-body-weight infants, starting with the first feed and continued until discharge, induced positive effects in necrotizing enterocolitis prevention. The early administration of selected probiotic strains improved the clinical outcome of pouchitis. The timing of probiotic administration in acute pathologies is crucial and may greatly influence the clinical outcome. One of the main targets of probiotic intervention studies is the restoration of GI microbiota composition and the correction of dysbiosis-associated markers of GI function. Probiotics are considered a therapeutic option in the management of inflammatory bowel disease (IBD). They may be administered as single organism or a defined mixture, with the aim of beneficially modifying the microbial ecology of the gut and GI symptoms. There is evidence that this treatment can prevent pouchitis and some evidence of benefit in the maintenance and treatment of ulcerative colitis (UC). In chronic IBDs, prolonged administration (range from 2 to 12 months) seems to improve the clinical outcome as demonstrated by the remission rate of mild to moderate UC in both children and adults. The duration of various treatments ranged from 6 to 12 weeks for the induction of UC remission, and 12 months for maintenance treatment. Multistrain probiotic VSL#3 treatment was also effective for the maintenance of remission of chronic relapsing pouchitis for 9 months or until relapse. The administration of a single strain of lactobacilli to patients with acute pouchitis for 1 or 3 months showed no effects, while in patients with mild active pouchitis, it induced partial improvement. There is no clear evidence to support the use of probiotics in Crohn's disease. The minimum duration of intervention in irritable bowel syndrome (IBS) patients was thought to be 4 weeks; prolonging treatment maintained the initial partial positive effect without further clinical improvement. As no curative treatments are available, therapy for IBS is palliative and supportive, targeting specific symptoms, but is notoriously unsatisfactory. Prolonging of treatment seems maintain the initial partial positive effects without further improvement of the intestinal conditions. *Conclusions:* The strain characteristics and appropriate dosage could contribute to improve their effects with an appropriate schedule of administration, according to the different etiologies of the GI disorders. Probiotics contribute to ameliorating GI symptoms along with standard therapy. The differences in host response to probiotic intervention need to be carefully evaluated.

Keywords: probiotics administration; diarrhea; inflammatory bowel diseases; clinical outcome

### Application of Tigecycline and Other Antibiotics Against Clinical and Environmental Isolates

#### **Bezirtzoglou, E.**^1^, Mantzourani, I.^1^, Panopoulou, M.^2^, Theodoridou, I.^1^, Tsirogiannis, I.^3^, Papaemmanouil, V.^3^, Johnson, B.^4^, Biedenbach, D.^4^, Bouchillon, S.^4^
^1^Faculty of Agricultural Development, Department of Food Science and Technology, and Laboratory of Microbiology, Biotechnology and Hygiene, Democritus University of Thrace, Alexandroupolis, Greece; ^2^Laboratory of Clinical Microbiology, School of Medicine, Democritus University of Thrace, Alexandroupolis, Greece; ^3^Microbiological Laboratory, “Metaxa” Anticancer Hospital, Piraeus, Greece; ^4^International Health Management Associates, Inc., Schaumburg, Illinois, USA

*Introduction:* The irrational and extensive use of antibiotics has led to an increase in the number of resistant strains of bacteria. As the study of new antibiotic agents is critical, tigecycline was investigated in this research survey. *Materials and Methods:* The activity of 11 antibiotics against 264 pathogens isolated from clinical specimens and 225 pathogens isolated from environmental samples (surface waters) was evaluated. Study groups included *Escherichia coli*, *Enterobacter* spp., *Enterococcus faecium* and other *Enterococcus* spp., *Klebsiella oxytoca* and other *Klebsiella* spp., *Pseudomonas* spp., and *Staphylococcus aureus*. Isolates were tested by the broth microdilution method under common concentrations and the results interpreted according to Clinical Laboratory Standards Institute (CLSI) criteria. *Results:* Up to 38% of environmental *E. coli* was resistant to amoxycillin/clavulanic acid, 65% of *S. aureus* to levofloxacin, and 39% of *Klebsiella* spp. and 33% of *Klebsiella oxytoca* to ceftriaxone, while 50% of environmental *E. coli*, 35% of *Enterobacter* spp., and 23% of *Pseudomonas* spp. showed resistance against cefepime. Results demonstrated that 27% of *E. coli*, 8% of *Enterococcus* spp., and 62% of *S. aureus*, all isolated from the environment, were resistant to tigecycline. *Discussion:* Our results indicate dispersion of antibiotic resistance into the environment. Comparison of similar strains reveals significantly higher total resistance to the environmental ecosystem than to clinical ecosystems. It is obvious that there is a rapid spread of new antibiotics in the environment as far as tigecycline is concerned, which has been used in Greece only in clinical practice for the last 7 years.

Keywords: environmental strains; clinical strains; antibiotics; susceptibility; tigecycline

### Occurrence and Antibiotic Susceptibility Profile of *Streptococcus* spp. Isolated from Ewe's Milk

#### **Bezirtzoglou, E.**^1^, Alexopoulos, A.^1^, Plessas, S.^1^, Abas, Z.^1^, Lagka, V.^2^, Zdragas, A.^3^
^1^Democritus University of Thrace, Greece; ^2^Alexander Technological Educational Institute of Thessaloniki, Greece; ^3^NAGREF Veterinary Research Institute of Thessaloniki, Greece

*Introduction:* Mastitis caused by bacteria resistant to various antimicrobial agents, is a major factor affecting milk quality. Mastitis in ewes, goat, or bovines can be caused by many different bacterial species, the most common of which are *Staphylococcus* and *Streptococcus*. *Materials and Methods:* The aim of this study was to determine the occurrence and antimicrobial resistance of *Streptococcus* spp. isolated from fresh ewe's milk. A total of 130 milk samples were collected from bulk tanks, analyzed for the presence of *Streptococcus* spp., and the isolates tested against 10 antimicrobial agents using the disk diffusion method. *Results: Streptococcus* spp. were observed in 84.4% of the samples, with counts ranging from 0.6 to 4 log CFU/ml (average: 3.5±3.7 log CFU/ml). The majority of samples (30%) had levels from 3 to 3.6 log CFU/ml. Only 1.3% of the isolates were resistant to all antimicrobials, while 27.3% were susceptible. Resistance to cefepime was most common (93.1%), followed by resistance to ampicillin (82.1%), ciprofloxacin (58.3%), and meropenem (51.9%). Vancomycin susceptibility was seen in 83.6% of isolates. Multidrug resistance to at least three antibiotics was observed in 22.1% of *Streptococcus* spp*. Discussion:* The occurrence of *Streptococcus* spp. was higher than reported by others but with log counts of the same order of magnitude. Values of overall resistance indicate that the isolated bacteria have significant resistance levels to at least four antibiotics. Considering this finding in conjunction with the elevated counts of streptococci in the vast majority of our samples, further examination and intervention is required.

Keywords: antibiotic; ewe's milk; *Streptococcus* spp.

### The Role of Gut Microflora in the Pathogenesis of Chronic Diseases and Possibilities for its Modulation for their Prevention

#### **Bomba, A**., Strojný, L., Chmelárová, A., Hijová, E., Bertková, I., Mojžišová, G., Žofčáková, J., Salaj, R., Supuková, A., Šoltésová, A. Institute of Experimental Medicine, Faculty of Medicine, Pavol Jozef Šafárik University in Košice, Slovakia

Chronic diseases are the most serious health problem in human medicine. Cancer and coronary heart disease are the most important disorders that cause high mortality and morbidity in humans. For many years, atherosclerosis and cancer were considered to have completely unrelated pathogeneses and disease progression pathways requiring separate therapeutic strategies. Current knowledge suggests that atherosclerosis and carcinogenesis shared many common features including predisposing and etiological factors and molecular pathways in pathogenesis. This could lead to the development of common therapeutic strategies. It may be hypothesized that common therapy approaches and preventive strategies could be efficacious in both diseases. Gastrointestinal microbiota play a very important role in health maintenance and disease prevention in humans. Signals from the intestinal microbiota are important for normal host physiology. A high fat diet adversely affects the gut microbiota and causes dysbiosis. The increase in dysbiosis causes low-grade inflammation and metabolic disorders. Dysbiosis is associated with a number of infectious and chronic diseases such as obesity, type 2 diabetes, atherosclerosis, and cancer. Human colonizing microbiota are essential to health, but the composition and functional characteristics of a healthy microbiome remain to be defined. Elucidation of the properties of healthy microbiota would provide a target for dietary interventions and microbial modifications aimed at sustaining health in generally healthy populations and improving the health status of people exhibiting disrupted microbiota and associated diseases. Current knowledge shows that sophisticated modulation of gut microflora using functional foods, probiotics, prebiotics, and natural bioactive substances could effectively decrease health risks. The results of our experiments using animal experimental models of atherosclerosis and chemically induced colorectal cancer, showed that probiotics and prebiotics beneficially influence the composition of gut microflora, its metabolic activity, gut barrier function, production of anti-inflammatory cytokines, antioxidative status, NF-κB activation, COX-2 and iNOS immunoreactivity, and lipid metabolism including reduction in plasma total cholesterol, LDL-cholesterol, and triglycerides, depending on the characteristics of the probiotic strain. The results of animal experiments and clinical studies indicate that probiotics could influence the common molecular pathways of both cancer and atherosclerosis pathogenesis. From that point of view, we suggested a new definition of probiotics as follows: “Probiotics are live microorganisms which modulate the specific function of organisms by activation of specific molecular pathways”. Future research should be aimed at discovering new and effective combinations of probiotics and natural bioactive substances in the form of dietary supplements or functional foods that will be able, through a combination of different mechanisms, to influence the pathogenesis of chronic diseases at different levels and molecular pathways, and to decrease the disease risk.

This work was supported by the Agency of the Slovak Ministry of Education for the Structural Funds of the EU, under project ITMS: 26220220152.

Keywords: gut microbiota; chronic diseases; prevention; probiotics; prebiotics

### Novel Composite Antimicrobials in Medicine and in the Agri-Food Chain

#### **Boyko, N.V.**^1,2^, Melnyk, V.^1^, Bati, V.^1^, Levchuk, O.^1^, Mizernytskyy, A.^3^, Spivak, N.^4^
^1^Uzhhorod National University, Ukraine; ^2^Cassovia Life Sciences, Slovakia; ^3^LCL “SGP MBS” Ltd, Kyiv, Ukraine; ^4^LCL “Diaprof”, Kyiv, Ukraine

*Introduction:* Multidrug resistant microorganisms (MDRM) are common in clinical units and contaminate the environment and food. The problem is compounded by dramatic changes in typical microbial communities marked by the prevalence of potentially pathogenic species which replace normal biota representatives by spreading MDR genes. A search for smart composite materials with strongly defined and industrially proved properties might be a key issue in the prevention of the dissemination of MDRM in medicine and the food chain. *Methods:* In order to screen for MDRM, an ESBL test was performed for all potentially pathogenic microorganisms and food-borne pathogens isolated from different sources: air, water, edible plants, soil, raw material, foods, animals, humans, and hospitals. Edible plants and berries rich in biologically active compounds together with selected probiotic bacteria and nanoparticles of cerium dioxide were meticulously tested for their antimicrobial properties *in vitro*, *in vivo*, and *in situ* against chosen MDRM. *Results:* After all screening experiments were conducted, the most effective and completely synergic compositions of selected plants extracts, probiotic strains, and carrier molecules (nanoparticles and chitosan) acting strongly specifically against targeted MDRM (namely, methicillin resistant *Staphylococcus aureus* (MRSA), *Streptococcus mutans*, *Proteus mirabilis*, *Escherichia coli* (EPEC), *Salmonella enterica*, *Pseudomonas aeruginosa*, *Klebsiella pneumoniae* subsp. *oxytoca*, and *Listeria monocytogenes*) were developed. No composition includes any components which can have an effect on normal typical commensal biota of each separately defined niche, except those which demonstrated their stimulating properties. The synergetic properties of all the components in the preparations most promising for implementation had been tested in a pilot study and the optimal ratios of components determined. The synbiotic preparation ‘ProFiLactOr’ is based on lactic acid bacteria strains due to their synergistic effect with plant carriers, and can be used for the normalization of mouth cavity and gut microbiota in children with caries and gastrointestinal disorders. The preparation's effect is unique due to its ability to selectively inhibit the growth of the main causative agents of periodontal tissue inflammation and intestinal colitis. The production technology of ‘Enteronormin’ and ‘Enteronormin-Detox’ is original and protected by patent. Strains used for the production of ‘Enteronormin’ are lodged in the depository of the Institute of Microbiology and Virology of NASU. The preparation is produced as a powder, whose main advantage is that it can be used as a suspension for oral hydration or as a 6% solution for aerosol treatment to disinfect feeds and buildings, as well as for external use, for example, for the prophylaxis and treatment of mastitis, vaginitis, and endometritis in animals. *Conclusions:* The synbiotic preparation ‘ProFiLactOr’ showed a strong antagonistic effect against indicators of decay and periodontal disease in children in a limited clinical trial. Two novel antimicrobial composite preparations, ‘Enteronormin’ and ‘Enteronormin-Detox’, were developed using classic biotechnological approaches, certified according to the state registration procedure, and applied in the livestock sector. An interventional study to evaluate nanoparticles as components for dressings promoting wound healing and burns is in progress. Currently, we are focusing on attempts to use novel antimicrobials which can specifically inhibit food-borne pathogens in the mitigation of biofilm formation.

Keywords: MDRM; edible plants extracts; novel composite antimicrobials; synbiotics

### The Effect of the Herbal Feed Additive Digestarom^®^ Used as a Dietary Supplement on the Chemical and Microbial Composition of Feces in Lactating Sows

#### **Brestenský, M**., Nitrayová, S., Patráš, P., Heger, J. Animal Production Research Center Nitra, Slovakia

*Introduction:* Herbs and herbal additives are known for their antimicrobial, antioxidant, and antifungal properties. Some of these compounds have been reported to improve animal performance because of their stimulating effect on pancreatic enzyme secretion or their direct bactericidal effect on gut microflora. The aim of this study was to examine the effect of plant product Digestarom^®^ used as a dietary supplement on the chemical and microbiological composition of feces in lactating sows. *Material and methods:* Twelve sows (Large White×Landrace, initial body weight 222.1±11.8 kg) were used in a 48 day study to evaluate the effect of the herbal feed additive Digestarom^®^ used as a dietary supplement at 0.15 g/kg, on the chemical and microbiological composition of feces. The sows were fed the standard diet for lactating sows supplemented (experimental group; n = 6) or not (control group; n = 6) with Digestarom^®^. Before parturition, the sows were fed 3 kg/day, and after parturition 4.8–7.2 kg/day, depending on the number of piglets per litter. Water was offered *ad libitum*. The animals were housed individually in a laboratory with a climate-controlled environment. The experiment started on day 20 before the planned parturition of sows. The feces were collected directly from the rectum on day 10 before planned parturition, and on day 2 and day 14 after parturition. The samples of feces were analyzed for dry matter, crude protein, and coliform, anaerobic bacteria, and *Salmonella* spp. content. *Results and discussion:* No significant differences were observed dry matter (DM) content in feces between the experimental and control group. The consistency of feces was the same in both groups, and diarrhea was not observed. The content of crude protein in the experimental group was insignificantly (*p*>0.05) higher (185.3 vs. 172.4 g/kg DM) on day 10 before parturition and insignificantly lower on day 2 (172.2 vs. 181.9 g/kg DM) and on day 14 after parturition (177.3 vs. 187.4 g/kg DM), indicating better utilization of crude protein in the control group on days 2 and 14. Microbial parameters in the control and experimental groups, especially the number of coliforms and anaerobic bacteria in feces, were not significantly affected by Digestarom^®^ added to the diets. All feces samples were negative for the presence of *Salmonella* spp. *Conclusions:* The herbal feed additive Digestarom^®^ added to the diets of lactating sows at 0.15 g/kg had no significant effect on the chemical and microbiological composition of feces. This article was written during realization of the project ‘ZDRAVIE no. 26220220176’ supported by the Operational Programme Research and Development financially supported by the European Regional Development Fund.

Keywords: Digestarom^®^; faeces; microbiota; plant extract; sows

### Nutrimetabonomics to Link Gut Microbial Modulations to Metabolic Health

#### Claus, S. University of Reading, UK

The gut microbiota is now recognized as a fundamental partner of the host's health. Nutrimetabonomics is a useful tool to assess the metabolic state of the host in response to gut microbial modulation. This method was successfully applied in mouse models to examine how the progressive colonization of the gut impacts on the host metabolism. Recently, this was also used in investigation of the effects of a prebiotic on obese patients and revealed positive correlations between bacteria of the genus *Propionibacterium* and increased levels of circulating VLDL, lactate, and phosphatidylcholine. Levels of *Collinsella* were also positively correlated with urinary hippuric acid. Preliminary results in a cohort of elderly patients showed that an increased level of lactobacilli was associated with enhanced concentrations of short chain fatty acids, lactate, and various amino acids in feces. More precisely, the presence of *Lactobacillus helveticus* was reflected in higher levels of glucose and lactate in the feces, suggesting that this probiotic may be of clinical interest in this population. These data indicate that nutrimetabonomics can be a powerful tool to study host–gut microbial interactions, which is of high interest for clinical applications and for developing personalized nutrition.

Keywords: nutrimetabonomics; elderly; lactobacilli

### The Effects of Different Antibiotics on Salivary Microbiome Profiles Determined by 16S rRNA Gene Amplicon Sequencing

#### Crielaard, W. Academic Centre for Dentistry Amsterdam, University of Amsterdam, The Netherlands

*Introduction:* The human body is colonized by a vast number of microbes: the human microbiome. The highest densities of microbes are found in the oral cavity and the gastrointestinal tract. A well-balanced microbiome is essential for the human host. In the current study we aimed to determine the effect of different antimicrobials on the ecological balance of the indigenous human oral microbiome. *Methods:* Salivary samples were collected from healthy individuals during clinical studies at two centers (Helperby and Karolinska), before (baseline), immediately after (week 1) antibiotic (minocycline, amoxicillin, ciprofloxacin, and clindamycin) or placebo administration, and 1, 2, 4, and 12 months later. Microbial DNA was extracted and quantified. Barcoded amplicon libraries of the small subunit ribosomal RNA gene hypervariable region V5–V7 were generated for each of the individual samples. These were pooled and sequenced by means of the Genome Sequencer FLX Titanium system. The sequencing data was processed using QIIME version 1.5.0. Quality filtered reads were denoised (Denoiser version 1.3.0) and chimeric reads identified (UCHIME version 4.2.40). The cleaned reads were clustered into operational taxonomic units (OTUs) at a minimal sequence similarity of 97% using the UCLUST Optimal clustering algorithm and the taxonomy assigned using the RDP classifier. Phylogenetic and diversity analyses were performed on randomly subsampled datasets. *Results:* Microbiomes from the placebo groups showed stable species richness and diversity throughout the year of the study. Samples obtained from the same individual showed high phylogenetic relatedness. Baseline and week 1 samples from the same individual in the placebo groups were highly similar. Some fluctuation in microbial composition was observed between more distant timepoints. Samples from individuals exposed to antibiotics (all but amoxicillin) showed reduced species richness directly after the administration of antibiotics (week 1) and a fast recovery of microbiome diversity (day 30) thereafter. Amoxicillin did not show any effect on species richness or microbiome diversity after the antibiotic administration period. However, this antibiotic was associated with strong individual responses, resulting in increased phylogenetic distances between the baseline and week 1 samples compared to the other groups (Helperby placebo and minocycline). Administration of minocycline, ciprofloxacin, or clindamycin resulted in distinct qualitative changes in the microbiome at week 1 (clusters of the week 1 samples in unweighted UniFrac plots), while on day 30 no difference from the baseline was observed. Certain taxa and phylotypes (OTUs) showed significant shifts in relative abundance after the administration of antibiotics. *Discussion:* During 1 year, the undisturbed (placebo) microbial ecology of the human oral cavity displays a very high degree of stability. Considering all the daily challenges that the system undergoes (food intake, temperature challenges, oral hygiene), we can conclude that this is a very robust ecosystem. Only extreme challenges, like the antibiotics used in our study, have a profound impact on the oral ecology. Surprisingly, the misbalance introduced by minocycline, ciprofloxacin, clindamycin, or amoxicillin was counteracted within 30 days after administration. Besides being very robust, the oral microbiome is also shown to be a very resilient ecosystem.

Keywords: microbiome; oral cavity; antibiotics; ecological impact

### Membrane Composition Changes of *Staphylococcus aureus* in Response to a Membrane-Perturbing Antimicrobial Peptide

#### **Desbois, A.P** ^1^, Young, S.A^2^, Coote, P.J^2^, Smith, T.K^2^
^1^University of Stirling, UK; ^2^University of St Andrews, UK

*Introduction: Staphylococcus aureus* is an important human pathogen that causes serious infections and many strains are resistant to multiple antibiotics which compromises clinicians’ ability to treat patients. To date, relatively little is known of the *S. aureus* membrane and its role in defending the cell against the actions of antimicrobial agents. *Methods:* In this present study, GC-MS was used to characterize the cell membranes of three diverse *S. aureus* strains, including Mu50 (an unusual vancomycin-intermediate resistant isolate), Newman (a well-studied methicillin-susceptible strain), and BB270 (a clinical isolate with laboratory-introduced methicillin resistance). *Results:* The fatty acids (FAs) present in the membranes of these strains contained between 14 and 20 C atoms, but BB270 completely lacked C19 and C20 FAs. Branched-chain FAs dominated the FA profiles of the three strains, especially iso-C15, anteiso-C15, and anteiso-C17. The ratios of straight-chain to branched-chain FAs were 4.6, 10.6, and 9.2 for BB270, Newman, and Mu50, respectively. Membrane composition was also characterized for two strains after serial passage in the presence of a membrane-perturbing antimicrobial peptide (AMP), ranalexin. During 20 passages, the minimum inhibitory concentrations (MICs) of the strains against ranalexin increased from 16–32 mg/l to >256 mg/l. The membranes of the resistant *S. aureus* strains contained more straight-chain FAs and lower proportions of branched-chain FAs. The mean length of the straight chain FAs were also increased in the resistant strains. These composition changes would decrease the fluidity of the membrane and may explain the decreased susceptibility of the cells to the antimicrobial action of ranalexin, as the AMP would be less able to integrate into its target. *Discussion:* This study demonstrates that membrane composition changes can reduce susceptibility to AMPs, which has implications for the use of these agents in clinical therapy and in innate immunity.

Keywords: antibiotic resistance; antimicrobial peptide; fatty acid; lipid analysis; mass spectrometry; *Staphylococcus aureus*

### Activity of Probiotics on Biofilm-Growing Pathogens of The Oral Cavity

#### Donelli, G. IRCCS Fondazione Santa Lucia, Italy

The ability of probiotic species to inhibit the growth of different oral pathogens has received much interest in the last decade, even though the inhibition mechanisms are not fully understood. In the healthy state, plaque biofilm and the surrounding tissues establish a relationship that, when balanced, contributes to maintaining mouth health. However, in the most common oral diseases, such as dental caries, mucositis, periodontal diseases, and peri-implantitis, potentially pathogenic microorganisms can influence this homeostasis, so gaining a predominant position within the oral biofilms and causing severe alterations in the oral ecology. In this way, the ‘healthy’ dental plaque transforms into a ‘pathogenic’ biofilm. For example, when subgingival microflora shifts from its prevalent composition of Gram-positive bacteria to high concentrations of Gram-negative obligate anaerobes, such as *Aggregatibacter actinomycetemcomitans*, *Porphyromonas gingivalis*, *Prevotella intermedia*, *Prevotella melaninogenica*, *Streptococcus intermedius*, and *Treponema denticola*, different oral diseases, including periodontitis, can arise. In recent years, the use of probiotics to fight pathogenic microorganisms in order to restore a ‘healthy’ oral microbiome, has been considered a promising adjuvant in the treatment of infectious processes of the oral cavity. As regards counteracting biofilm-growing oral pathogens, a small number of experimental data on the effectiveness of probiotic microorganisms have been reported so far. In fact, the anti-biofilm effect of probiotics on anaerobic pathogens of the oral cavity was assessed in a few *in vitro* studies and mainly evaluated against streptococci. In light of the pivotal role played by *Lactobacillus* spp. in stimulating natural immunity, in producing anti-inflammatory effects, and in contributing to the balance of microflora by interacting with other species, the mechanisms involved in the reduction of the bacterial load of oral pathogens by lactobacilli have been recently investigated. However, further investigations on probiotic microorganisms able to compete for the adhesion sites present in the mouth and/or to produce antimicrobial substances capable of inhibiting the proliferation of oral pathogens, are required. Our laboratory has recently contributed to this promising research area by demonstrating the ability of *Lactobacillus brevis* CD2 to interfere with the development and maturation of *Prevotella melaninogenica* PM1 biofilm, both in the presence of the culture supernatant and all the *L. brevis* cells, thus suggesting the underlying inhibition mechanisms have a multifactorial nature.

Keywords: probiotics; biofilms; inhibition mechanisms; *Lactobacillus brevis*; *Prevotella melaninogenica*

### Genetic Structure and Cytotoxic Potential of *Bacillus cereus sensu lato* from northeastern Poland

#### **Drewnowska, J.M**., Święcicka, I. University of Bialystok, Poland

*Introduction:* Members of the *Bacillus cereus sensu lato* group persist in different ecological environments and have a huge impact on human health and economy. This group includes *Bacillus thuringiensis* commonly used as a bio-insecticide, *Bacillus cereus* which is associated with food-borne illness, and *Bacillus anthracis*, the anthrax agent. Despite numerous studies focused on these bacilli and examining species affiliation, the ecological diversification of *B. cereus s.l*. remains largely undescribed. *Methods:* Multi-locus sequence typing (MLST) was used to assess the genetic structure and phylogeny of 273 soil *B. cereus s.l*. isolates from highly diverse habitats in northeastern Poland: (i) the last European natural forest from which all human activity is totally excluded (Bialowieza National Park), (ii) the largest marshes in Europe (Biebrza National Park), and (iii) arable land in Jasienowka. Additionally, the ability to grow at low temperature and cytotoxic potential measured by the presence of the *cytK* gene and relative expression (RT-PCR), was analyzed in order to assess the ecotypic structure of *B. cereus s.l. Results:* The study revealed high genetic diversity of the isolates in seven housekeeping loci (325 alleles) which pertained to 148 sequencing types (STs; 131 new and 17 already described in the MLST database). The ratio of non-synonymous to synonymous mutations (*dN/dS*) was less than 1 for all loci in all isolates, indicating purifying selection within the genes. The goeBURST approach allowed the isolates to be grouped into 19 complexes corresponding with bacterial clones, and 80 singletons. Phylogenetic analyses showed that 74% of the isolates clustered with *B. cereus s.l*. environmental references (clade III), while only 11% and 15%, respectively, grouped with isolates of clinical origin (clade I), and *B. cereus* ATCC 14579 and reference *B. thuringiensis* (clade II). Predominantly within clade III, we found lineages adapted to low temperature (thermal ecotypes), while cytotoxigenic potential (*cytK*-positive) was intermixed among all clades of the marshy and farm samplings and is not associated with species affiliation. *Discussion:* The results of our study provide new insights into the population structure of *B. cereus s.l*. The occurrence of 92% of STs in bacilli originating from one habitat, and the description of new STs for 78% of the isolates, strongly indicate the existence of specific genotypes within the natural *B. cereus s.l*. populations. In contrast to the human-associated *B. cereus s.l*., the environmental isolates appear more intricate and diverse. Based on the genetic properties of the isolates, we did not find strong arguments for grouping the particular species into one taxon. Thus, we propose dividing *B. cereus s.l*. into two groups, the first including environmental isolates, and the second covering those of clinical relevance. In addition, the presence of *cytK* was mainly associated with isolates from farm samplings where human activity is extensive, while the gene was absent in isolates originating from Bialowieza National Park. Moreover, the *cytK* presence was intermixed among the lineages and species. This supports the opportunistic pathogenicity model of *B. cereus s.l*., where the potential or ability to cause various diseases has no association with specific pathotypes.

Keywords: *Bacillus cereus*; genetic structure; MLST

### Lactobacillus Curbs Inflammatory Responses to *Atopobium vaginae*

#### **Fichorova, R.N**., Moses, R.P., Fashemi, T., Yamamoto, H.S., Dawood, H.Y., Delaney, M.L., *Onderdonk, A.B*.
Brigham and Women's Hospital and Harvard Medical School, USA

*Introduction:* Bacterial vaginosis (BV) is a common infection in women of reproductive age, and is most commonly associated with over-population of the female lower genital tract by *Gardnerella vaginalis* or *Atopobium vaginae*. A healthy female lower genital tract is exemplified by dominant colonization by hydrogen peroxide-producing *Lactobacilli* species (i.e. *L. acidophilus* and *L. crispatus*), which may help prevent infections by suppressing opportunistic bacteria. Disruption of the vaginal mucosa provides favorable conditions for the onset of BV. Information about the interaction between *Lactobacillus* and *Atopobium*, and its relationship to BV is limited. In this study, immortalized human epithelial endocervical cells were used to examine the effect *of L. acidophilus* (La), *L. crispatus* (Lc), and *A. vaginae* (Av) in the female lower genital tract. *Methods:* We examined the effect of each bacterium in individual and mixed monolayer co-cultures. After a 24 h anaerobic incubation, innate immunity mediators were measured to compare epithelial cell responses. Antibiotic-free culture medium was used as the control. Supernatants were collected by means of electrochemiluminescence, and Elafin by means of ELISA. Lysate was collected for determining colony forming units (CFUs) and measuring NFκB activity via luciferase concentration. CFUs were obtained by parallel inoculation of Brucella anaerobically (non-selective) and Rogosa aerobically (selective against *Atopobium*). The GraphPad Prism 5, two-way ANOVA Bonferroni multiple comparison test, was used for statistical analysis of all results. Statistical significance was defined by a *p*-value of <0.05. *Results:* Activation of NFκB was triggered by all bacteria. In contrast, levels of the proinflammatory chemokine IL-8 were increased by more than a factor of 15 in Av-treated epithelia, while either *Lactobacillus* species caused no significant deviation. IL-8 levels released by epithelial cells treated with a mixture of La and Av or Lc and Av were increased relative to no bacteria control but decreased relative to Av-treated cells, despite similar or lower total CFU counts. Levels of RANTES, which serves as a bridge between innate and adaptive immunity, were similarly altered by bacterial co-culture. Elafin is a bactericidal anti-inflammatory protein, upregulated by mucosal injury and inflammatory insults. Elafin concentrations were elevated by Av alone. *Discussion: Lactobacillus* spp. are capable of suppressing the epithelial inflammatory response to *A. vaginae*, which may improve the prognosis of BV.

Keywords: bacterial vaginosis; *Atopobium vaginae*; lactobacillus; innate immunity; polymicrobial interactions

### Determination of the Antimicrobial Activity of Microbicide-Containing Soap Formulations against Skin Bacteria

#### **Forbes, S.**^1^, Latimer, J.^1^, Sreenivasan, P.^2^, Masters, J.^2^, McBain, A.^1^
^1^The University of Manchester, UK; ^2^Colgate-Palmolive Technology Center, Piscataway, NJ, USA

*Introduction:* The skin microbiome is a polymicrobial community that colonizes superficial layers of the healthy human skin and includes commensal organisms and adventitious pathogens. This investigation determined the effects of washing the skin with commercially available liquid or bar soaps formulated with antimicrobials or without antimicrobials on the resident skin flora. *Methods:* Human volunteers in good general health (>18 years of age) were enrolled after ethics board approval. For each subject (four adults, age range 20–35 years), the regions of both forearms from the wrist to the elbow were delineated into five distinct sample areas. The microbiota from both forearms was collected for microbiological evaluation via skin swabbing prior to washing with either a liquid or bar soap, representing a baseline sample. Subsequently, each arm was washed once for 30 s with either a test or control formulation. The test liquid and bar soaps were formulated with 0.15% or 0.4% triclocarban, respectively, while corresponding controls did not contain antimicrobials. Post-wash samples were collected from each arm immediately after washing and at 2 h intervals for up to 6 h. Subjects were instructed not to use other antimicrobial containing soaps, lotions, or cosmetic formulations for the duration of the study. All samples were evaluated for microbial viability by plating dilutions onto enriched agar. The microbial viability of samples was further determined using the AlamarBlue dye reduction assay, whereby viable microorganisms incubated in the presence of AlamarBlue reduce the blue dye to a red color, as determined spectrophotometrically. *Results:* While significantly lower numbers of viable skin bacteria were recovered immediately after washing with all formulations in comparison to baseline values (*p*<0.05), the use of either of the antimicrobial-containing test formulations achieved significantly larger reductions in bacterial viability, in comparison to their corresponding controls (*p*<0.05). Furthermore, significantly lower levels of viable skin bacteria were detected for up to 6 h post-washing with the test formulations in comparison to the non-antimicrobial containing controls (*p*<0.05). The AlamarBlue viability assay demonstrated significant differences between the test and control formulations at each post-wash time point (*p*<0.05). *Discussion:* Results from this investigation demonstrate significantly greater reductions in viable resident skin bacteria after one wash with an antimicrobial-containing liquid or bar soap than could be observed when using the corresponding formulations without antimicrobial adjuncts. Residual antimicrobial effects of the test soaps lasted longer than after the corresponding control formulations.

Keywords: skin; microbiota; antimicrobial

### Fecal Microbiota in Post-Weaning Piglets Fed ZnO or Humates with ZnO as Dietary Supplements Studied by 454-Pyrosequencing

#### **Kaevska, M**., Videnska, P., Trckova, M., Moravkova, M. Veterinary Research Institute, Czech Republic

*Introduction:* The post-weaning period is critical in piglets’ lives as they are then at their most susceptible to enteric disease. Since the banning of antibiotic use in the EU, different food supplements have been used to decrease the susceptibility of young piglets to enteropathogenic bacteria. The aims of our study were to determine the microbial diversity in piglets’ feces using high-throughput sequencing, and to detect changes in piglets fed ZnO or humates with ZnO as supplements. *Methods:* The post-weaning piglets at 28 days of age used in this study were fed ZnO or humates with ZnO as supplements during a period of 3 weeks. We investigated the bacterial composition of the intestinal microflora in three groups of piglets: group 1 received ZnO (2.5 g/kg of feed), group 2 received a mixture of sodium humate (HNa, 20 g/kg feed) and ZnO (Zn 1.39 g/kg feed), and a control group (group 3) was fed on a standard diet without any supplements. Each group consisted of three animals. The first analysis was done on fecal samples immediately after weaning and before the animals were given dietary supplements. The second analysis was performed after 3 weeks on the respective diets. Fecal DNA was isolated using a commercially available kit (Stool DNA Isolation kit, Qiagen, Germany) and 16S rDNA was amplified using universal bacterial primers targeting the variable regions V3 and V4. The amplified products were sequenced on the 454 GS Junior platform from Roche, according to the recommended protocol. Sequences were analyzed using QIIME software. *Results:* After quality checking, trimming, and chimera elimination, there were approximately 15,000 sequences for each group and for each sampling. The indigenous microflora consisted mainly of Bacteroidetes and Firmicutes, with lower levels of Actinobacteria, Fusobacteria, and Proteobacteria. There were only minor changes in microbial composition on a family level between the first and the second sampling in all groups. We recorded an increase in the abundance of the families *Bacteroidaceae* and *Enterobacteriaceae*, which was more notable in group 1. Also, there was a decrease in *Lactobacillaceae* and *Streptococcaceae* in all groups. The presence of *Fusobacteriaceae* after 3 weeks was recorded only in group 1 (12% of the microbiota). *Discussion:* Generally, the microflora in piglets’ feces was not affected dramatically by the ZnO or humates mixed with ZnO used as supplements. The overall increase in microbial diversity after 3 weeks following weaning could be attributed to normal development rather than changes caused by the dietary supplements. The most notable change was the presence of Fusobacteriaceae in group 1 after 3 weeks on the diet supplemented with ZnO.

Keywords: pyrosequencing; 16S rRNA; diet supplements; microbiota

**Acknowledgments**

This work was supported by grants from the Ministry of Agriculture (No. MZe0002716202 and QJ1210112) and the Ministry of Education, Youth and Sports (AdmireVet, CZ 1.05/2.1.00/01.0006; ED0006/01/01) of the Czech Republic.

### Comparative Study of the Lifestyle of Students of the Technical University in Košice and the Faculty of Medicine at P.J. Šafárik University in Košice

#### **Kimáková, T**., Čarnogurská, D., Korchňáková, M. Faculty of Medicine, Pavol Jozef Šafárik University in Košice, Slovakia

*Introduction:* Lifestyle is an important factor influencing human health; however, recently unhealthy lifestyles are found not only in adults but increasingly in children and teenagers. This study monitored recognized risk factors such as unhealthy nutrition, insufficient physical activity, stress, smoking, and consumption of alcohol and drugs in two groups of students: one group had been educated in risk factors and the other had not. We present the findings from a questionnaire on lifestyle in a selected set of respondents and compare the results with those of earlier research. *Methods:* The study was carried out from November to December 2012. The research cohort consisted of medical students from the Faculty of Medicine, P.J. Safarik University in Košice (LF) and non-medical students from the Faculty of Building Industry and the Faculty of Metallurgies, Technical University in Košice (TUKE). A total of 287 students aged 18–30 years were enrolled and the response rate was 100%. *Results and discussion:* Research results indicate the presence of many risk factors in the lifestyle of college students, which can impact on their future health. The most serious risk factors found were alcohol consumption, smoking, a total lack of physical activity, poor nutrition, and frequent consumption of sweets and fast food in both observed groups. Presumably, the risk factors negatively affect the balance of intestinal microflora, which, as it regulates several organism functions, may have a negative effect on overall health. The LF group of students showed a lower percentage of smokers, lower daily consumption of sugary drinks and alcohol, less physical activity, and higher sensitivity to noise compared with the entire cohort of monitored respondents. The TUKE students showed higher consumption of alcoholic beverages and tobacco products, but also a higher frequency of physical activity. Obesity and overweight as determined by body mass index (BMI) was three times more prevalent in male TUKE students than in the LF students. In contrast, underweight students were twice as common in the LF female group than in the TUKE female group. The statistical significance of BMI differences in the female and male groups (*p*-test) was less than 0.002. In the TUKE group, high blood pressure as a related risk factor (over 140/90 mmHg) was found in 42.3% (n=58) of male students and 6% of female students (n=10). Low blood pressure was found in 16% (n=18) of female LF students. *Conclusions:* Our research showed that previously published data describing the positive impact of education on maintaining a healthy lifestyle, were not replicated in our well-educated respondents. We expected that medical students, who should be aware of health risk factors, would achieve far better results regarding healthy lifestyles compared to technical students, who are not taught about risk factors and their impact on lifestyle.

Keywords: life style; risk factors; health

### Effects of Arginine on Microbial Ecological Shifts in Oral Microcosms

#### **Koopman, J.E**., Buijs, M.J., Crielaard, W., Zaura, E. ACTA, The Netherlands

*Introduction:* The oral cavity is a complex ecosystem, and its highly diverse microbial community comprises bacteria, Archaea, fungi, viruses, and protozoa. In a healthy host, there is a balance between the microbes and the host itself. Perturbations such as frequent exposure to fermentable carbohydrates, may disturb this balance, resulting in an ecological shift towards a diseased state. Potential prebiotics such as the amino acid arginine, may have a beneficial effect by increasing the environmental pH, thus inhibiting microbial shifts. The aim of the present study was to assess the effects of an arginine supplement on oral microcosms under highly cariogenic growth conditions. *Methods:* The eight-station multi-plaque artificial mouth (MAM) model was inoculated with plaque-enriched saliva from a healthy volunteer. Four of the stations received defined mucin medium (DMM) supplemented with arginine (1.6% w/v) (DMM + ARG). The remaining four stations were exposed to DMM without arginine (DMM-ARG). To mimic cariogenic challenges, all stations received eight 6 min sucrose (10% w/v) pulses a day at 2 h intervals. Each day contained a ‘resting period’ of medium alone (DMM + ARG or DMM-ARG) lasting 10 h. Organic acid anions (formate, succinate, acetate, lactate, propionate, and butyrate) in resting and fermenting biofilms were measured using capillary ion electrophoresis. The ammonium concentration in the biofilms was measured using an enzymatic ammonium assay. The biofilms were sampled at 14 time points (t) divided over 22 days, including eight time points where the biofilms were ‘at rest’, four time points following a 6 min sucrose pulse, and two time points following an arginine (8% w/v) pulse. Extracted DNA from the biofilms was used to assess Candida abundance through RT-PCR and to obtain a bacterial ecological profile through denaturing gradient gel electrophoresis (DGGE). *Results:* Biofilms exposed to arginine had a higher ammonium concentration compared to those not receiving additional arginine (2.96±0.45 and 2.04±0.44 mM/µl). The acid profiles showed that the DMM-ARG biofilms had produced significantly more butyrate and acetate (0.58±0.55 and 1.06±1.02 µmol/mg protein) than the DMM + ARG biofilms (0.17±014 and 0.60±0.33 µmol/mg protein), irrespective of the metabolic state. Candida abundance increased with time and was generally higher in the DMM-ARG biofilms, (t1: DMM+ARG 2.1×10^3^ CFU/ml, DMM-ARG 6.3×10^3^ CFU/ml; t14: DMM+ARG 2.0×10^4^ CFU/ml, DMM-ARG 2.7×10^5^ CFU/ml). Additionally, DGGE of the DMM-ARG and DMM+ARG biofilms resulted in clearly distinguishable profiles for both conditions. *Discussion:* Arginine reduced the acid challenge induced by sucrose, inhibiting outgrowth of Candida and preventing a shift in microbial composition towards a more aciduric and acidogenic community. The actual compositional changes will be investigated using next generation sequencing approaches in the near future. Based on these preliminary findings, arginine has a strong potential in stabilizing the oral ecosystem during cariogenic perturbations.

Keywords: arginine; oral; microcosms

### Assessment of the *In Vivo* Immunomodulatory Effect of Non-Viable Components of Probiotic *Enterococcus faecium* Culture Stimulated with Heat-Inactivated Cultures of *Escherichia coli* and *Bacillus cereus*

#### **Lazar, V.**^**1**^, Ditu, L.-M.^1^, Chifiriuc, C.^1^, Bezirtzoglou, E.^2^, Marutescu, L.^1^, Bleotu, C.^3^, Pelinescu, D.^1^, Mihaescu, G.^1^
^1^Faculty of Biology, University of Bucharest, Romania; ^2^Faculty of Agricultural Development, Democritus University of Thrace; ^3^Institute of Virology “St. Nicolau”, Bucharest, Romania

*Introduction:* The interaction of viable probiotic bacteria with other species from intestinal microbiota involves competitive exclusion phenomena (competition for nutrients and adhesion sites) and the synthesis of inhibitory molecules. The interaction with the human host occurs regardless of probiotics’ viability and is mediated by the interaction of bacterial cells/sub-cellular fractions with mucosal associated lymphoid tissue structures, activating both non-specific and specific immune defense. *Purpose:* The purpose of this study was the quantification of the serum levels of pro and anti-inflammatory interleukins in holoxenic mice infected with *Pseudomonas aeruginosa* before and after oral administration of non-viable fractions of the probiotic *Enterococcus faecium* CMGB 16 strain stimulated with heat-inactivated cultures of *Escherichia coli* and *Bacillus cereus. Material and methods:* Probiotic *E. faecium* CMGb 16 culture, grown in the presence of heat-inactivated cultures of *E. coli* O28 and *B. cereus* for 24 h at 37°C, was subsequently separated into supernatant (SN) and heat-inactivated cellular sediment (CS) fractions by centrifugation. The immunomodulatory properties of the obtained non-viable components (NVC) were tested on holoxenic mice. Each NVC was orally administered in three doses, at 24 h, before (as a prophylactic method), and after the experimental infection with *P. aeruginosa* (as treatment method). Blood samples were collected from the retinal artery by the non-traumatizing retro-orbital puncture method in the inner corner of the eye. The serum concentrations of IL-6, IL-1α, and IFNγ interleukins were assessed by the enzyme linked immunosorbent assay (ELISA) method, using a mouse ELISA kit (Thermo Scientific)*. Results:* Infection with *P. aeruginosa* after the prophylactic administration of non-viable probiotic components increases serum levels of IL-6 and IFNγ. The NVC obtained by growing the probiotic strain *E. faecium* CMGB 16 in the presence of *B. cereus* heat-inactivated culture induced a rapid increase in the IL-6 serum level of mice infected with *P. aeruginosa*. The inflammatory stimulus represented by bacterial infection potentiated the immunostimulatory effect of probiotic components on the synthesis of IL-6. The same effect was observed for IFNγ, especially after the prophylactic administration of CS of the probiotic strain *E. faecium* CMGB 16 grown in the presence of heat-inactivated culture of *B. cereus* and *E. coli* O28. Therapeutic administration of NVC after infection of holoxenic mice with *P. aeruginosa* causes increased serum levels of IL-1α, while *P. aeruginosa* infection after NVC prophylaxis lowers the level of this cytokine. *Conclusions:* Probiotic bacteria produce molecules that interfere with the cytokine signaling pathways of the host organism, suggesting their involvement in the modulation of the host anti-infectious defense ability, mediated by cytokines able to activate the non-specific (IL-1, IL-6) or specific (IFNγ) immune reactions, that is, attenuation of the inflammatory response by increasing IL-6 (which induces acute phase protein synthesis), activation of a specific immune response reflected in increasing levels IFNγ, and limiting inflammatory bowel lesions by lowering the IL-1α level.

Keywords: probiotic non-viable components; serum cytokines; mice

### Biofilm Tolerance and New Anti-Biofilm Strategies Based on Traditional Antimicrobial Natural Products Containing Quorum Sensing Inhibitors

#### Lazar, V. University of Bucharest, Romania

The increased cellular density of a microbial community or biofilm and the consequent accumulation of signaling or quorum sensing (QS) molecules helps the metabolic activity of microbial cells in this new growth mode, including their tolerance to antimicrobials and synthesis of virulence factors, adapt better by synchronizing their expression of different genes at a particular cell density, according to each step of the infectious process. The prognosis of these chronic infections, compared with a metastatic phenomenon, following biofilm development on cellular substrata and biomaterials used in medicine, is worsened by a particular property – behavioral resistance or *tolerance*, even though the component cells tested in suspension (by the standard method) may be susceptible to some antibiotics. In this context, the research to find and test new preventive or therapeutic anti-infectious strategies has become a top scientific priority. Progress in the study of biofilms has opened a new perspective in research on new antipathogenic drugs, without any selection pressure and induction of resistance or other side effects, based on the discovery that the success of pathogens is due to their ability to sense the environment through signals concerning their own density and also signals produced by different cells of the associated or host organisms. Therefore, they must be able to receive signals and to respond to them, activating their virulence genes and tending to avoid or inactivate the host's unspecific and specific defense mechanisms and to use the host factors for their own benefit. It has been shown that many organisms, such as commensal bacteria (and probiotics), algae, lichens, plants, and animals synthesize molecules called QS inhibitors (QSI) as an anti-infectious defense mechanism which can interfere with the QS mechanism of pathogens and reduce their capacity for harm, as an expression of their co-evolution and co-adaptability. In recent years, following the general trend of using different drugs provided by nature, plant and bee products have been investigated and promoted as providing a wide range of harmless therapeutic applications. Plants are well-known for containing different antimicrobials, including QSI; more recently, it was shown that honey bees can protect themselves against pathogens by producing antimicrobials and QSI. Interkingdom signaling is a relatively recent field of research, which holds promise for efficient anti-infectious therapy management. The aim of this lecture is to present the main pathways for combating pathogens and reducing the expression of virulence factors using natural antimicrobials targeting the QS circuits, focusing on bioactive molecules extracted from plants and honey bee products. Scientists do not intend to use QSI or other active molecules exclusively, but to combine them with active antibiotics. It is an intelligent strategy to harness the ability of QSI to interfere with the QS mechanism, thus rendering biofilms susceptible to normal doses of antibiotics.

Keywords: microbial biofilms; tolerance to antimicrobials; anti-biofilm strategies; quorum sensing; natural QS inhibitors

### Infection Impact of Two *Brachyspira pilosicoli* Strains on the Metabolic Profile of Chicken Feces

#### **Le Roy, C.I.**^1^, Mappley, L.J.^1^, La Ragione, R.M.^2^, Woodward, M.J.^1^, Claus, S.P.^1^
^1^University of Reading, UK; ^2^University of Surrey, UK

*Brachyspira pilosicoli* is one of the main pathogens responsible for avian intestinal spirochetosis (AIS). AIS is responsible for much financial loss due to reduction in weight gain and in egg quality and quantity. In order to better understand the local metabolic impact of AIS on chicken microbiota, this study compared the effects of two strains of *B. pilosicoli* (B2904 and CPSp1) on experimentally challenged commercial laying chickens. Fecal samples were collected before and after infection for monitoring using a ^1^H-NMR-based metabonomic approach coupled with multivariate analysis. The results demonstrated a number of metabolic variations between the control and both challenged groups, which were consistent with symptoms such as weight loss and increased fecal moisture content. Infection by CPSp1, which was arguably more pathogenic *in vivo* and caused greater pathology, was correlated with an elevated fecal glucose content, indicating a decrease in intestinal glucose adsorption potentially related to weight loss in the chickens. This result is key to understanding some of the symptoms associated with *B. pilosicoli* infection.

Keywords: AIS; *Brachyspira pilosicoli*; chicken; metabonomic

### Conserved Molecular Mechanisms Used to Degrade Heteroxylans in Gut Bacteroidetes

#### **Mackie, R.I.**^1,3^, Dodd, D.^2,3^, Hong, P.^1^, Zhang, M.^3^, Cann, I.K.O.^1,2,3^
^1^Department of Animal Sciences, University of Illinois, USA; ^2^Department of Microbiology, University of Illinois, USA; ^3^Institute for Genomic Biology, University of Illinois, USA

*Introduction:* Enzymatic depolymerization of dietary fiber by bacteria in the human colon is critical to gut health and function. Initially, to identify the mechanism utilized by *Prevotella bryantii* B_1_4 to degrade xylan, a rumen bacterium that efficiently hydrolyzes and ferments hemicellulose, the transcriptomes of *P. bryantii* cultured on either wheat arabinoxylan or a mixture of its monosaccharide components, were compared by DNA microarray and RNA sequencing approaches. The most highly induced genes formed a cluster that contained putative outer membrane proteins analogous to the starch utilization system identified in the prominent human gut bacterium *Bacteroides thetaiotaomicron*. The arrangement of genes in the cluster was highly conserved in other xylanolytic Bacteroidetes, suggesting that the mechanism employed by xylan utilizers in this phylum is conserved (Dodd et al. J Biol Chem 2010; 285: 30261–73; Dodd et al. Mol Microbiol 2011; 79: 292–304). We predicted that this approach would provide a functional understanding of the mechanisms involved in degradation and utilization of hemicellulose by prominent colonic Bacteroidetes. *Methods:* To extend and confirm this finding in human colonic Bacteroides, we selected *Bacteroides ovatus* ATCC 8483 and *Bacteroides intestinalis* DSM 17393 for further study. Each strain was cultured on either wheat arabinoxylan (WAX) or xylose. The growth rate of *B. ovatus* was 0.55 and 0.29 h^−1^ on xylose and WAX, respectively. The growth rate of *B. intestinalis* was slower at 0.24 and 0.14 h^−1^ on xylose and WAX, respectively. Each strain grows at the same rate on glucose, xylose, and arabinose. Cells were harvested at mid-log, and total RNA was extracted and submitted for RNA-Seq analysis using the Illumina HiSeq 2000 platform. *Results:* Bioinformatic analysis was used to predict GH encoding genes to examine if the arrangement of genes involved in xylan degradation is conserved among the examined Bacteroidetes. The mRNA of an endoxylanase of unusual modular organization was found to be the most highly upregulated transcript, and transcriptional analyses found similar results with *B. ovatus* and a rumen *Prevotella*. Examination of the publicly available databases suggested that related genes are almost exclusively found in the Bacteroidetes. Among the top 12 highly induced genes in *B. intestinalis* cells fermenting WAX, were transcripts that encode novel combinations of GH catalytic modules to optimize hemicellulose degradation. Using both biochemical and biophysical approaches, we probed the architecture of the components of these hemicellulolytic enzymes to provide insights into the strategies evolved by a human gut bacterium to harvest nutrients from fiber. Furthermore, a similar array of transcripts observed in *B. ovatus* grown on the same substrate suggests that this strategy is conserved in other Bacteroidetes that metabolize hemicellulose in the human gut. *Conclusions:* The transcriptomic studies show that the most highly induced genes formed a cluster of genes termed the xylan utilizing system (XUS) analogous to the starch utilization system (SUS) identified in *B. thetaiotaomicron*. The arrangement of genes in the cluster is highly conserved in the three examined xylanolytic Bacteroidetes, suggesting that the degradative mechanism employed by xylan degraders is also conserved.

Keywords: bacteroidetes; *B. intestinalis*; *B. ovatus*; hemicellulose; glycoside hydrolases

### Health of Roma Living in Roma Settlements

#### Madarasova Geckova, A. Pavol Jozef Šafárik University in Košice, Slovakia

The Roma are one of the largest and oldest minorities in Europe. The health of many of them, particularly those living in settlements, is heavily compromised by poor dwellings, low educational level, unemployment, and poverty rooted in generational poverty, segregation, and discrimination. The aim of the lecture is to describe and discuss selected aspects of health in the population living in Roma settlements using findings from recent surveys carried out in an area with a high concentration of this population (eastern Slovakia). The following surveys will be used: (1) ecological surveys on the effects of selected socio-economic factors and ethnicity on infant mortality, mortality in productive age, and alcohol-related mortality (2004, 2002, 2001–2003, respectively), (2) a cross-sectional survey on the prevalence of hepatitis B/C and cardiovascular disease risk factors (HepaMeta, 452 Roma and 403 non-Roma adults aged 18–55 years, 2011), and (3) a school-based cross-sectional survey on health and health-related behavior (330 Roma and 722 non-Roma adolescents, approximately 15 years old, 2007). Special attention will be paid to methodological challenges related to this kind of research. The proportion of inhabitants living in Roma settlements significantly contributes to regional differences in the infant mortality rate but not to the standardized mortality rate of the population of productive age, nor to the alcohol-related mortality rate. The population living in Roma settlements has a higher chance of accumulating cardiovascular risk factors compared with the major population, but differences in younger age groups were not as dramatic as the differences found in older age groups. The lower self-rated health among Roma in comparison to non-Roma subjects seems to be partially explained by their worse access to health services: Roma living in settlements perceived lack of money for medication and transport, and bad experiences, fear, or lack of trust to be their most common difficulties. The poorer health outcomes reported by Roma adolescents compared to non-Roma adolescents can be partially attributed to the lower socio-economic status of Roma parents, discrimination, and hopelessness, while on the other hand, parental social support might serve as a protective factor. Some regions are at risk of decline due to long-term problems with poverty, high unemployment, and depleted resources, while some populations are also at risk, in the case of Central and Eastern Europe mainly those living in Roma settlements, which restricts the potential for public health improvements and consequently economic prosperity. Therefore, we should devise appropriate strategies and tools (e.g. Roma mediators, field work), while also considering their long-term sustainability.

Keywords: Roma settlements; mortality; cardiovascular disease risk factors; self-reported health

### Microbial Ecology of The Male Genital Tract

#### Mändar, R.^1,2^
^1^University of Tartu, Estonia; ^2^Competence Centre on Reproductive Medicine and Biology, Estonia

Understanding of the human microbial communities continues to grow rapidly; a recent (July 2013) PubMed search using the term ‘microbiome’ revealed more than 4,200 publications. At the same time, characterization of the male genital tract communities has always lagged behind investigations in other body sites: ‘penis microbiome’ revealed seven publications, ‘urethra microbiome’ four, ‘coronal sulcus microbiome’ two, and ‘male genital tract microbiome’ one publication, while ‘semen microbiome’ produced no results. Nevertheless, the male genital tract microbiota has been researched by applying cultures and ‘old’ molecular methods for several decades. It is generally accepted that a microbiota exists in the lower male genital tract. The penis itself provides distinct anatomical environments in the urethra and coronal sulcus. Both sites provide a suitable microbiotope for aerobic, microaerophilic, and anaerobic bacteria. According to newer mass sequencing studies, the most frequent phyla in the urethra are Firmicutes, Actinobacteria, Fusobacteria, Proteobacteria, and Bacteroidetes, while high inter-subject variability exists. The coronal sulcus microbiota appears to be more stable than that of the urethra, but its composition is influenced by circumcision, which is associated with a significant decrease in anaerobic bacteria. The upper genital tract is believed to be germ-free, except in case of infections. Studies of prostate specific specimens have given contradictory results; however, several investigations indicate that prostatitis patients have frequently abundant polymicrobial communities in their semen, expressed in prostatic secretion and/or post-massage urine. Therefore, prostatitis has been sometimes considered dysbiosis of the genital tract. Many prostatitis-related bacteria appear to be biofilm producers or exhibit anti-complement activity. Prostate tissue is normally expected to be germ-free, however, studies have provided contradictory findings that may be the result of contamination during sampling, the focal nature of inflammation, or a very high prevalence of prostatitis in elderly men. The genital tract microbiota is significantly associated with male health. First, susceptibility to sexually transmitted diseases (STDs) may be associated with community type, since STD positive men have more fastidious, anaerobic, and uncultivated bacteria. However, the present data are insufficient to determine whether the STD-associated communities precede, are co-transmitted with, or are established subsequent to STD. Second, the transfer of microorganisms to the prostate gland may lead to several consequences since recent studies have shown that diseases of this organ are more closely associated than supposed in the past. Prostatitis that begins at a younger age can significantly interfere with complaints of benign prostatic hyperplasia and is an important risk factor for prostate cancer and/or infertility. Third, genital tract dysbiosis-related prostatitis is characterized by oxidative stress (OxS) – an imbalance between the production and detoxification of reactive oxygen species that can cause tissue damage. Prostatitis-associated local OxS is accompanied by systemic OxS. Since OxS is involved in the pathogenesis of many diseases such as cancers, cardiovascular, and even mental diseases, prostatitis-associated OxS may pose an increased risk for the development of these conditions.

Keywords: microbiota; male genital tract; urethra; coronal sulcus; prostate; impact on man's health

### The Genital Tract Microbiome in Infertile Couples

#### **Mändar, R.**^1,2^, Borovkova, N.^1,2^, Lapp, E.^1,2^, Korrovits, P.^1,2^, Punab, M.^1,2,3^, Metspalu, A.^1^, Krjutškov, K.^1^, N[otilde]lvak, H.^1,2^, Preem, J.^1,2^, Oopkaup, K.^1,2^, Salumets, A.^1,2^, Truu, J.^1,2^
^1^University of Tartu, Estonia; ^2^Competence Centre on Reproductive Medicine and Biology, Estonia; ^3^Tartu University Hospital, Estonia

*Introduction:* Genital tract microbial communities are tightly associated with reproductive function, and their imbalance may explain reproductive failure in some cases. It is highly likely that the male genital tract microbiota has an impact on the female microbiota. Previous studies have demonstrated an increase in vaginosis-type microbiota and coliforms in women, as well as adverse effects of male genital tract bacteria on *in vitro* fertilization and pregnancy outcome. We profiled the seminal and vaginal microbiome of infertile couples by applying Illumina sequencing. *Methods:* We investigated 23 infertile couples. Semen samples were collected during the menstruation of their partner. Two self-collected vaginal samples were taken on the 6th to 8th days of the menstrual cycle, before and 8–12 h after intercourse. Seminal and vaginal microbiomes were profiled by applying the method which uses combinatorial sequence tags attached to PCR primers that amplify the rRNA V6 region (967–985 and 1078–1061, *Escherichia coli* 16S rRNA segment). Amplified PCR products were sequenced using an Illumina paired-end protocol on the HiSeq 2000 platform. Overlapping segments of the forward and reverse reads were analyzed with SHERA software. Mothur software was applied to trim, screen, and align sequences, calculate distances, assign sequences to operational taxonomic units, and obtain distance matrices. *Results:* Semen samples were clearly divided into two groups based on community composition. One group was dominated by *Gardnerella vaginalis*, *Corynebacterium* spp., *Bifidobacterium breve*, *Peptoniphilus* spp., *Porphyromonas* spp., *Prevotella* spp., *Lactobacillus* spp., *Campylobacter rectus*, *Staphylococcus hominis*, Fam. *Marinilabiaceae*, Fam. *Comamonadaceae*, and some unclassified Proteobacteria and Firmicutes. The second group was dominated by *Enterobacteriaceae*, *Prevotella* spp., *Lactobacillus coleohominis*, *Lactobacillus iners*, *Diaphorobacter* spp., *Stenotrophomonas* spp., and some unclassified Firmicutes, Actinobacteria, and Proteobacteria. In men with inflammation in their semen (>1 M WBC/ml), the mean proportion of Proteobacteria was higher than in non-inflammation men (*p*=0.045). Most of vaginal samples were dominated by lactobacilli (mainly *Lactobacillus iners* and *Lactobacillus crispatus*), while a small proportion of women had *Gardnerella vaginalis* or other bacteria (*Streptococcus* spp., *Veillonella* spp., *Atopobium vaginae*, *Pseudomonas* spp., Fam. *Enterobacteriaceae*). Seminal communities were significantly more diverse than vaginal ones, but with lower bacterial concentrations. In the couples with more similar genital tract microbiota, the similarity decreased after intercourse, while in the couples with less similar microbiota, the similarity increased after intercourse. *Discussion:* According to our knowledge, this is the first study to compare the seminal and vaginal microbiome and the effects of intercourse on the latter by applying Illumina sequencing. At the same time, the weakness of the study is the absence of a fertile control group; however, this type of study is quite complicated and healthy couples are not sufficiently motivated to participate. We revealed that the microbiomes of semen samples of the male partner in infertile couples are less abundant but highly diverse, while those of vaginal samples are more homogenous. The impact of the seminal microbiome during intercourse caused surprising but significant shifts in the vaginal microbiome, with the similarity of couples being decreased when they are more different before intercourse, while similarity is increased in couples with lower initial similarity. Further studies should determine the functional meaning of these phenomena.

Keywords: seminal microbiome; vaginal microbiome; sexual intercourse; infertile couple

### In Vivo Spread of Macrolide-Lincosamide-Streptogramin B (MLSB) Resistance: a Model Study in Chickens

#### **Marosevic, D**., Cervinkova, D., Vlkova, H., Videnska, P., Babak, V., Jaglic, Z. Veterinary Research Institute, Czech Republic

The extensive use of antimicrobial agents in animal husbandry poses a risk for the selection of resistant microorganisms within the both pathogenic and commensal microbiota. The gastrointestinal tract of animals has already been defined as rich in a variety of resistance genes and such animals can serve as reservoirs of resistant bacteria or genetic determinants of resistance that can be transmitted to humans. In this study, 4-week-old chickens colonized with *Enterococcus faecalis* carrying pAMβ1 (the challenge strain) were perorally exposed to three different antibiotics (tylosin, lincomycin, and chlortetracycline) during 1 week. Cloacal swabs were taken at four different time points for bacteriological examination and isolates of resistant *E. faecalis* and *Enterococcus faecium* were subjected to pulsed-field gel electrophoresis (PFGE) and plasmid isolation. The *erm(B)* gene was quantified in the DNA isolated from feces during a 2-week period by qPCR. The isolated DNA was subjected to 16S-rDNA pyrosequencing in order to obtain better insights into the fecal microbiota composition and changes induced by the different antibiotics administered. A total of 77 enterococci and 22 streptococci were isolated, the majority of them originating from the TYL and TET groups during or after antibiotic administration. Two PFGE clones of each *E. faecalis* and *E. faecium* were observed, all different from the challenge strain. All isolates of enterococci and streptococci harbored a plasmid of the same size as that of pAMβ1. qPCR revealed an increase in *erm(B)* in all treated groups during the entire period of monitoring and in two out of five chickens from the non-treated negative control group at the last sampling point, whereas the challenge species *E. faecalis* rapidly declined over time. The relative composition of fecal microflora was dependent on the antibiotic administered and the sampling time point. In the current study, an increase in the *erm(B)* gene was observed among all treated groups. The impact of non-specific antibiotic pressure exerted by chlortetracycline was similar to that of tylosin and lincomycin; furthermore, in two out of five chickens from the negative control group, a significant increase in *erm(B)* was detected at day 42. This is an interesting finding, indicating that the spread of genetic determinants of MLS_B_ resistance may be independent not only of the specificity of antibiotic pressure but also of the antimicrobial pressure *per se*. A similar phenomenon was also observed under field conditions where MLS_B_ resistant bacteria were isolated from piglets which had never before been treated with macrolides or lincosamides. The pyrosequencing results obtained in this study do not supply exact information on the spread of MLSB resistance; however, as observed for enterococci, these correlated with the qPCR results. The *erm(B)* gene increased in all treated groups and, therefore, it could be speculated that those bacterial taxons which prevailed under certain conditions might be considered as potential recipients. Thus, it could be presumed that MLS_B_ resistance can spread in different ways depending on the particular antibiotic pressure.

Keywords: MLSB resistance; chicken; microbiome; tetracycline

### The Probiotic Concept: Will It Survive?

#### Midtvedt, T. Karolinska Institute, Sweden

Microbiologically fermented products, especially milk, have been ingested since ancient times, long before Élie Metchnikoff claimed in 1908 that bacteriologically fermented milk products could benefit health. The term ‘probiotics’ was first used half a century ago and is currently defined as live organisms, which when utilized in adequate amounts, exert a health benefit. In the last two to three decades, the production of probiotics has greatly increased world-wide, as has also the number of publications. Today, close to 10,000 articles are listed in PubMed, and the number is increasing monthly by approximately 100, covering fields such as human consumption, animal husbandry, pets, fish farming, etc. Most products for human consumption are based upon lactobacilli and/or bifidobacteria, while a large variety of species are used in animal and fish products. At our SOMED meeting in Yokohama 2 years ago, we discussed the rules regarding the acceptance of health claims world-wide. Here in Europe, producers still have some difficulties getting their health claims accepted. Personal opinion regarding future rules for the safe use of probiotics in humans suggests the following will be required: (1) genome characterization; (2) product characterization; (3) safety characterization; and (4) functional characterization. Each of these four points will be briefly commented upon. In the future, companies will supply product(s) with well-documented genetic and phenotypic properties, creating new niches for prophylactic and therapeutic use, and thereby fulfilling a statement by Hippocrates nearly 2500 years ago: “Let food be your medicine and medicine your food”.

Keywords: genome characterization; probiotic concept; health claims

### The Possibility of Applying Lactobacillus sp. Strains for Health in the Whole Food Chain

#### Mikelsaar, M. University of Tartu, Estonia

This review describes a versatile approach for improving the quality of the whole food chain by using beneficial lactobacilli from human microbiota and the environment. In the last 50 years, there has been an huge increase in the number of publications and related innovations. The lengthy search for beneficial bacteria, and the identification of these as probiotics or natural preservatives of plant materials, has resulted in biotechnological applications of the new strains for safe and healthy functional food and animal feed. Fermented dairy products are important foods containing high value proteins and bioactive compounds, but are unevenly available world-wide. There are serious deficiencies in the biotechnological handling of different materials such as legumes, grasses, and high-clostridia silages. Globally, serious outbreaks of food-borne infections have been reported, highlighting the need for better natural preservatives against the contamination and oxidation of food. At the same time, the prevalence of some chronic diseases such as atherosclerosis, rnetabolic syndrome, hypertension, and obesity, is clearly rising. The international organizations (WHO, UNESCO, ILSI, etc.) consider evidence-based nutrition crucial to help control such diseases. Therefore, new approaches to food production and nutrition science are vital to ameliorate the huge socio-economic load. The University of Tartu houses a large collection of human lactobacilli (HUMB at the Department of Microbiology, a member of the European Culture Collections’ Organisation). In 2004, the Biocompetence Centre of Healthy Dairy Products (BCCHDP) was established under the Estonian biotechnology program. The university and BCCHDP work together to create a synergy between agriculture, the food industry, and medicine. Estonian microbiologists have developed five novel probiotic food products, which are or are in the process of being patented and licensed globally. In the 1970s, the University of Tartu participated in the development of three *Lactobacillus* spp. used as probiotic strains for Russian astronauts against dysbacteriosis during space flights. In the late 1990s, an antimicrobial and antioxidative probiotic, *Lactobacillus fermentum* ME-3 (DSM 14241), was developed to reduce oxidative stress and metabolic risk factors in atherosclerosis, food allergy, and persistent intestinal infections. In the late 2000s, the team patented two *Lactobacillus plantarum* (DSM 21380 Tensia and DSM 21379 Inducia) strains and a multispecies combining *L. plantarum* MCC1 and *Lactobacillus gasseri* MCC2 as a probiotic functional food to improve human metabolism through its antihypertensive effects and reduce the risk of allergy by immunological effects. The dairy products containing the elaborated probiotic strains are naturally preserved against oxidation and contamination, thus guaranteeing their safety****. The environmental strain *L. plantarum* E-98 (NCIMB 30236) as a natural technological additive for obtaining good-quality feed, is authorized by the European Food Safety Authority and carried into EU registry. The developed products are considered protective against infections and contamination in dairy products. These natural fermented products can be transported all over the world to developing countries with food shortages and lacking valuable proteins.

Keywords: lactobacilli; probiotics; application for whole food chain; natural preservation of dairy food and animal feed; efficacy; prevention of metabolic diseases

### Lactobacillus Plantarum Biocenol^TM^ LP96 and n-3 PUFAs and their Immunomodulatory Action

#### **Mudroňová, D**., Nemcová, R., Chytilová, M., Gancarčíková, S., Koščová, J., Tkáčiková, L’., Lazar, G. University of Veterinary Medicine and Pharmacy, Slovakia

Probiotic lactobacilli as well as n-3 PUFAs are well-known immunomodulators. In our previous experiment performed in conventional weaning piglets, we noted positive effects on clinical status, the composition of gut microbiota, and the cellular immune response after the application of probiotic lactobacilli and crushed flaxseed. In the subsequent trial, we studied the mode of immunomodulatory action of flaxseed oil (rich in n-3 PUFAs) and the probiotic strain *Lactobacillus plantarum* Biocenol^TM^ LP96 in gnotobiotic pigs infected by enterotoxigenic *Escherichia coli* (ETEC). Eighteen germ-free piglets were obtained via open hysterotomy and divided into four groups: group K (control, n=3), group L (*Lactobacillus plantarum*, n=5), group MK (flaxseed oil, n=5), and group LMK (*L. plantarum*+flaxseed oil, n=5). The piglets from the LMK and L groups were inoculated orally each day with 2 ml of the *L. plantarum* strain (1×10^9^ CFU/ml). Flaxseed oil was supplemented to the animals in groups LMK and MK daily at a dose of 0.5 ml. At the age of 5 days, the piglets from all groups were challenged orally with 2 ml of *E. coli* O8:K88ab:H9:ent^-^ strain (1×10^5^ CFU/ml). Numbers of lactobacilli and *E. coli* were monitored in gastrointestinal content and adhering to the jejunal and ileal mucosa. The proportions of lymphocyte subpopulations in peripheral blood and jejunal mucosa as well as phagocytic activity were measured by flow cytometry. Gene expression levels of selected cytokines and TLR were analyzed by qRT-PCR. Our results indicate that adhesion of *L. plantarum* to jejunal and ileal mucosa as well as the counts of lactobacilli in the intestinal content were markedly promoted by flaxseed oil. When compared to the control and L groups, the counts of *E. coli* K88 adhering to the jejunal and ileal mucosa were decreased significantly in the LMK animals. *L. plantarum* also showed potency to increase the percentage of CD4+ lymphocytes in the intraepithelial compartment, since flaxseed oil increased the CD8+ subpopulation in the intraepithelial compartment as well as in the jejunum lamina propria. In all experimental groups, we noted a higher percentage of regulatory T lymphocytes (CD4 + CD25 + ) as compared to control. Our results showed that combined treatment down-regulates IL-1α and IL-8 gene expression (which correlates with down-regulation of TLR2, TLR5, TLR9, and transcription factor NF-κB), up-regulates IFNγ, and tends to regulate inflammation induced by ETEC through cytokine IL-10. The stimulatory effect of PUFAs on lactobacilli adhesion suggests that they could be used for enhancing the effectiveness of probiotics in inhibiting digestive tract pathogens. In general, changes in cytokine gene expression correlated with the proportions of immune cells isolated from the same part of the jejunal mucosa and with the expression of TLR. This publication is the result of project No. 26220220152 implementation supported by the Research and Development Operational Programme funded by the European Regional Development Fund.

Keywords: immunomodulation; probiotic lactobacilli; flaxseed, *E. coli*; pig model

### An Optimal Framework for Chronic Condition Management in Europe

#### Nagyova, I.^1,2^

#### ^1^Institute of Public Health, Department of Social Medicine, Graduate School KISH, Faculty of Medicine, Safarik University, Košice, Slovakia; ^2^European Public Health Association

*Issue/problem:* European countries have achieved major improvements in public health over recent decades. Life expectancy at birth in the EU-27 countries has increased over the last 50 years by a decade. The European population is ageing rapidly, but living longer does not necessarily mean living a healthier, more active, and independent life. Indeed, some Europeans are living to just over 60 before poor health or disability affects their lives. Chronic diseases are the main reason for poor health and restricted activity, affecting over one third of Europe's population. Many chronic conditions and diseases are linked to an ageing society, but also to lifestyle choices such as smoking, alcohol consumption, exercise, and diet, as well as to genetic predisposition. Whether or not longer life expectancy is accompanied by good health and functional status among ageing populations has important implications for health, social, and long-term care systems. *Description:* Given this background, policy-makers across Europe are increasingly searching for interventions and strategies to tackle chronic disease in those with complex health needs, and are initiating new models of service delivery. The key lies in promoting people-oriented, demand-driven innovations in smart investments that have the potential to meet the needs of the changing demographic environment. A variety of changes in the management of chronic disease care have been advocated. Most effective interventions for improvements in chronic disease care include the combination of multi-pronged strategies. The Chronic Care Model (CCM) and the Expanded CCM are examples of such strategies. Both strategies foster systemic change, with the CCM focusing on people who have a disease and the Expanded CCM also supporting people and communities to be healthy. *Lessons:* The primary objective of this presentation is to raise awareness of the current situation in the field of chronic condition management in Europe and to highlight some good practice examples to showcase the value and importance of working within a chronic condition management framework. The outcomes of several research projects dealing with the management of chronic conditions will be presented: (1) the EPPOSI project highlighting the main existing gaps and weaknesses in the management of chronic conditions across 10 EU countries; (2) the LORIDISS project on mixed self-management programs for chronic disease care across five chronic diseases; and (3) the EMBRACE project evaluating the cost-effectiveness of the combined CCM and population-health management model. *Conclusions:* Collaborative, integrated, and people-centered care provision is a way forward for sustainable and efficient care systems. The success of the CCM and its contribution to support the necessary changes in current healthcare systems depends on the active involvement of, and collaboration among, a broad range of committed stakeholders.

Keywords: chronic condition management; chronic diseases; public health; multidisciplinary approach; integrated care

### The Pig as a Valuable Model for Monitoring Digestion and the Ecosystem of the Digestive Tract

#### **Nitrayová, S**., Brestenský, M., Heger, J., Patráš, P. Animal Production Research Center Nitra, Slovakia

*Introduction:* The use of pigs in research, specifically as a preclinical model, has increased dramatically since the early 1980s. Similarity to humans in size, physiology, and genetics makes the pig an excellent model for many organs and systems. Cannulated pigs allow the study of nutrient digestibility, live tissue sampling, the insertion of compounds or therapeutic agents into a specific region of the gastrointestinal tract, and also investigation of the dynamics of intestinal microflora. The aim of this study was to prepare various types of cannulated pigs as a model for nutrition research and to choose the most suitable type of cannulation. *Material and methods:* During the last 8 years we have implemented and optimized the following digestive tract cannulas: T-cannula, re-entrant cannula, and post-valve T-cecum cannula. Using these devices, we successfully cannulated and maintained 95 pigs with body weights ranging from 9.5 to 60.0 kg. *Results and discussion:* Simple T-cannulation was performed successfully on 75 pigs and we consider it an optimal cannulation procedure. An incision was made beginning approximately 7 cm caudal to the last rib and continued parallel to the left costal arch for about 13 cm. A T-cannula was inserted in the appropriate part of the intestine. A 1.5 cm hole was then bored caudal to the incision and the cannula was exteriorized through this hole to the surface of the body. We also prepared polycannulated pigs. Ten female pigs (35 kg bodyweight) were surgically modified, and one simple T-cannula was inserted into the jejunum and a second into the terminal ileum approximately 10 cm cranial to the ileo-caecal valve. Polycannulated pigs were used in a project studying the impact of probiotics and prebiotics on the morphological and functional parameters of the gastrointestinal tract. The post-valve T-cecum cannula was inserted between the ileum and the cecum and routed through the abdominal wall to the surface. When we prepared re-entrant cannulated pigs, the intestine was interrupted, and the cannulas were inserted in two sections of intestine and routed through the abdominal wall to the surface of the body. Intestinal chyme passes outside the body of an animal through a created bridge. All cannulated pigs recovered quickly following surgery and after 10–14 days they were eating normally. Elevated body temperatures were not seen in any pigs, and clinical problems related to surgery were not observed. *Conclusions:* The simple T-cannula will be used in our future experiments involving intestinal cannulation in pigs as a reliable tool when studying nutrient digestion and absorption and to check changes in the ecosystem of the gastrointestinal tract in animal as well as human studies. This article was written during implementation of project ‘ZDRAVIE no. 26220220176’ supported by the Research and Development Operational Programme funded by the European Regional Development Fund.

Keywords: animal model; cannulated pigs; surgery; digestive tract

### The Distribution and Evolution of Antibiotic Resistance Genes in Coagulase-Negative Staphylococci (CoNS) Isolated from Volunteers Treated and Not Treated with Antibiotics

#### **Perreten, V.**^1^, Endimiani, A.^2^, Collaud, A.^1^, Hu, Y.^3^, Coates, A.^3^, Rashid, M.-U.^4^, Weintraub, A.^4^, Strauss, Ch.^1^
^1^Institute of Veterinary Bacteriology, University of Bern, Bern, Switzerland; ^2^Institute of Infectious Diseases, University of Bern, Bern, Switzerland; ^3^Helperby Therapeutics, London, UK; ^4^Division of Clinical Microbiology, Department of Laboratory Medicine, Karolinska Institutet, Karolinska University Hospital, Huddinge, Sweden

Coagulase-negative staphylococci (CoNS) are major inhabitants of the normal skin and nasal flora. They have been used as indicator organisms to determine the distribution and evolution of antibiotic resistance genes in volunteers who received minocycline, amoxicillin, clindamycin, or placebo. Healthy volunteers (10 in each group) who had not been treated with antibiotics for at least 3 months received minocycline, amoxicillin, clindamycin, or placebo for 10 days. Nasal and skin samples were taken before antibiotic administration, as well as immediately after, 1 month, 2 months, and 12 months post-dosing. Nasal and skin samples were screened for the presence of CoNS on blood agar plates. Species were identified using MALDI-TOF MS. The antibiotic resistance genes were detected using a microarray platform capable of detecting up to 115 antibiotic resistance genes known among Gram-positive bacteria. *Staphylococcus epidermidis* in the placebo group volunteers was characterized by MLST. CoNS were found to harbor antibiotic resistance genes conferring resistance to beta-lactams (*blaZ*, *mecA*), aminoglycosides (*aac(6)-Ie–aph(2)-Ia*, *aph(3)-III*, *ant(6)-Ia*, *ant(4)-Ia*), tetracyclines (*tet*(M), *tet*(K), *tet*(L)), macrolides, and lincosamides (*erm*(C)) in all volunteer groups. The number of *tet*(M)-containing staphylococci increased in the group of volunteers treated with minocycline compared to placebo and other treatment groups. No increase in beta-lactam resistance genes *blaZ* and *mecA* was observed in the group of volunteers who received amoxicillin. Similarly, no increase in lincosamide resistance genes was observed in the group of volunteers who received clindamycin. In particular, four out of five subjects in the placebo group were colonized with isolates carrying the *mecA* gene. Volunteers were either colonized with the same type of methicillin-resistant *S. epidermidis* strains over the year or with several types of methicillin-resistant *S. epidermidis* strains. The use of amoxicillin and clindamycin did not lead to an increase in antibiotic resistance genes in CoNS from volunteers. On the other hand, minocycline clearly selects for a *tet*(M)-containing Staphylococcus flora. Of note, healthy people can harbor in their nose and skin methicillin-resistant CNS isolates with an extended assortment of antibiotic resistance genes. The clinical impact of such a reservoir of genes remains to be evaluated. The research leading to these results has received funding from the European Union Seventh Framework Programme (FP7/2007-2013) under grant agreement no. 241446.

Keywords: staphylococcus; methicillin; antibiotic resistance; genotyping

### Identification of Factors Influencing *Staphylococcus aureus* Resistance, Fitness, and Virulence

#### **Senn, M.M.**^1^, Dengler, V.^1^, Quiblier, Ch.^1^, Berger-Bächi, B.^1^, McCallum, N.^1,2^
^1^Institute of Medical Microbiology, University of Zurich, Switzerland; ^2^Centre for Infectious Diseases and Microbiology, Westmead Hospital, Sydney, Australia

*Introduction:* Methicillin resistance in *Staphylococcus aureus* can be influenced by factors outside the resistance island SCC*mec* (staphylococcal cassette chromosome *mec*), which encodes the resistance determinant penicillin binding protein 2a (PBP2a). PBPs are involved in the last steps of peptidoglycan synthesis, an essential component of bacterial cell wall. PBP2a is active in the presence of concentrations of beta-lactams blocking the endogenous PBPs and abolishing cell wall biogenesis in methicillin-sensitive *S. aureus*. The level of methicillin resistance, fitness, and virulence can be modulated by factors involved directly or indirectly in cell wall synthesis. As part of the ANTIRESDEV project, we identified several new factors affecting these properties. *Methods:* Transposon-mutant library screening, transcriptome analysis, whole genome sequencing, and virulence assessment using animal models were performed. *Results:* (1) The new regulator XdrA, influencing methicillin resistance levels and homogeneity, as well as growth rate, was shown by transcriptomic analysis to also affect gene expression, including transcription of one of the most prominent immunomodulatory *S. aureus* proteins, SpA. (2) The two-component system VraSR, involved in stress response to cell wall active antibiotics and influencing resistance towards these antibiotics, was further characterized. A new player, YvqF, was identified to physically interact with VraS and to influence resistance. (3) Studies on the LytR-CpsA-Psr proteins of *S. aureus* showed the importance of these factors for resistance, fitness, and virulence. Furthermore, experiments supported a role of these proteins in the synthesis of wall teichoic acids, cell envelope constituents influencing cation homeostasis, cell separation, and colonization of the human host. (4) Analysis of a transposon-mutant library identified 17 new genes modulating beta-lactam resistance, and in some cases also affecting growth rate, cell envelope stability, response to cell wall stress, and virulence factor expression. (5) Comparison by whole genome sequencing of a faster-growing, but less methicillin-resistant variant compared to its parent, revealed a single nucleotide polymorphism in the diadenylate cyclase gene *dacA*. DacA synthesizes the second messenger cyclic diadenosine monophosphate that has been proposed to be involved in cell envelope homeostasis. Re-introduction of the mutation into the highly resistant but slower growing parent strain reduced resistance and increased the growth rate, suggesting a direct connection between the *dacA* mutation and the phenotypic differences of these strains. *Discussion:* Numerous new factors influencing the resistance, virulence, and vital properties of *S. aureus* were identified, pointing to physiological processes previously unknown to be important for resistance. Unraveling how exactly all these factors can modulate such diverse properties and tasks such as sensitivity towards antibiotics, maintenance of fitness, or expression of virulence, requires further work. Importantly, these genes have the potential to reduce the biological cost caused by antibiotic resistances and to allow resistant strains to maintain considerable fitness and virulence, making them interesting targets for the development of much required new antimicrobial substances.

Keywords: *Staphylococcus aureus*; methicillin; resistance; virulence; fitness

### Nutritional and Gut Microbiota Programming of Health and Diseases

#### Shenderov, B.A. G.N. Gabrichevsky Moscow Research Institute of Epidemiology and Microbiology, Russian Federation

Many modern chronic metabolic diseases are strongly related to human nutrition patterns and the composition of indigenous gut microbiota. Both these environmental factors play a critical role in the epigenomic programming of human long-term health, well-being, and risk of metabolic diseases. The term ‘programming’ describes the process through which exposure to the lifestyle of pregnant women (diet, physical activity, occupation, income, bad habits), as well as environmental exogenous (medicines, pathogenic microbes, various pollutants) and endogenous (indigenous host microbiota composition) factors and agents during pregnancy and the nursing period, can stimulate or suppress fetal and newborn growth and development, resulting in permanent functional and metabolic changes *in utero* and in offspring, increasing susceptibility to disease later in the infant, child, adolescent, and adult. Epigenetic alterations in fetus chromatin and histones may be the mechanisms playing key roles in the person's life and might be inherited and affect the health of future generations. In this report, the author discusses the main epigenomic mechanisms of programming perturbations caused by under- or over-nutrition and/or host microecology imbalance in the pregnant woman and infant during the first 1000 days (from the start of pregnancy through to the second year of life), that are potentially capable of producing life-long unfavorable phenotypic consequences. Some approaches are offered regarding the design of various functional foods enriched with food ingredients and/or ingredients of microbial origin possessing metabolic and signal activity that can affect gene expression in the host metagenome and post-translation modification of gene products interfering in different epigenetic biochemical mechanisms.

Keywords: diet; indigenous gut microbiota; programming; epigenetics

### Rapid Microarray-Based Detection of Antibiotic Resistance Genes: an Improved DNA Labeling and Amplification System

#### **Strauss, Ch.**^1^, Endimiani, A.^2^, Collaud, A.^1^, Perreten, V.^1^
^1^Institute of Veterinary Bacteriology, University of Bern, Switzerland; ^2^Institute of Infectious Diseases, University of Bern, Switzerland

Over the last two decades, world-wide antibiotic usage in clinics, animal husbandry, and breeding has increased the abundance of antibiotic resistant strains in multiple bacterial genera. It is therefore essential to develop tools to rapidly detect resistance genes in hospitals and to enable the tracking back of resistance genes in all environments. To this purpose, there has been a great effort to reduce the overall costs and time required in the use of gene detection systems, including multiplex PCR analyses and microarray detection systems. Here, we describe a microarray-based detection system for antibiotic resistance genes in Gram-positive bacteria using a new labeling method. Combining essential steps such as DNA fragmentation, amplification, and labeling steps, this simplified method allows the simultaneous detection of 116 antibiotic resistance genes abundant in Gram-positive bacteria. The DNA was fragmented and amplified using phi-29 polymerase and random primers with linkers. Labeling and further amplification were then performed by classic PCR amplification using biotinylated primers specific for the linkers. A total of 244 oligonucleotides (26–37 nucleotides in length with similar melting temperatures) are spotted on the microarray (ArrayTubes, Alere), including the resistance genes and positive controls. The specificity of the oligonucleotides was verified by testing a collection of 73 Gram-positive strains which cover 95% of the antibiotic resistance genes detectable with the novel microarray platform. Several well-characterized multidrug-resistant strains of *Staphylococcus aureus*, *Enterococcus faecium*, *Lactococcus lactis*, *Clostridium perfringens*, and *Streptococcus salivarius* were analyzed and within a single reference *S. aureus* strain (BM3318), up to 19 genes could be detected. Furthermore, a total of 85 antibiotic resistance genes in 28 out of the 73 reference strains were detected for the first time using the new system. The improved DNA amplification and labeling system allows sample processing, from DNA amplification to the final results, to be carried out in a single working day. Finally, DNA labeling and amplification was shown to be independent of the input DNA and the microarray system used, which allows it to be adapted for Gram-negative bacteria as well as for virulence factors. The protocol described here offers a rapid, sensitive, and cost-effective approach for applications in basic research, food safety, and surveillance programs for antimicrobial resistance. This project has received funding from the European Union Seventh Framework Programme (FP7/2007-2013) under grant agreement no. 241446.

Keywords: antibiotic resistance; microarray; Gram-positive; rapid

### Health Effects of the Application of Probiotic Bacteria in Different Matrices

#### **Štšepetova, J.**^1^, Songisepp, E.^2^, Hütt, P.^1^, Mikelsaar, M.^1^
^1^University of Tartu, Estonia; ^2^Bio-Competence Center of Healthy Dairy Products LLC, Tartu, Estonia

Functional probiotic products are aimed to beneficially affect human metabolism and the functionality of the cardiovascular system. Probiotic bacteria expressing various functional properties have been consumed in various products with different food matrices and formulations. After consumption, the probiotic *Lactobacillus* sp. bacteria should temporarily colonize the targeted region of the gastrointestinal (GI) tract, block some adhesion sites on mucosa or mucus, and initiate metabolism. Metabolites absorbed into the bloodstream are predicted to influence the intestinal microbial ecology and reach different organ systems. *Aim:* Evaluation of the health effects of the probiotic strain *Lactobacillus fermentum* ME-3 (DSM14241) after consumption with different food matrices and food supplements. Beneficial changes in oxidative stress markers in the blood of participants enrolled in different registered probiotic trials were compared. *Material: L. fermentum* ME-3 has been elaborated for probiotic products such as yoghurt, kefir, and food supplements. *L. fermentum* ME-3 produces antimicrobial and antioxidative compounds (acetic, lactic, and succinic acids, ethanol, and Mn-superoxide dismutase) and harbors the entire glutathione system. The stability of the functional properties of ME-3 in food and food supplements was confirmed. Altogether, four probiotic food and food supplements efficacy trials were performed. The results of an open placebo-controlled fermented goat milk trial (n=21 persons) and randomized double-blind, placebo-controlled probiotic kefir trial (n=73) were compared with randomized double-blind, placebo-controlled probiotic capsule (n = 24) and randomized double-blind, placebo-controlled synbiotic (n = 53) trials. Daily doses of 3×10^11^, 4×10^10^, 3×10^9^, and 6×10^9^ CFU were administered for 3 weeks. Fecal counts (log CFU/g) of lactobacilli (LB) and the presence of the consumed strain (RAPD-PCR) were determined at baseline and after the intervention. The values of blood ox-LDL, baseline diene conjugates (BDC-LD), total antioxidative status (TAS), and ratio of glutathione/reduced glutathione (GSSG/GSH) were assessed. *Results:* Consumption of ME-3 increased the LB counts in all trials, while the strain was assessed in the fecal samples of participants only in food matrix trials. A reduction in ox-LDL, BCD-LDL, and the GSSG/GSH ratio was detected in all four trials irrespective of consumption dose. *Conclusions:* In principal, if a probiotic strain is confirmed to survive in gastric and gut milieu, and has increased the total count of fecal lactobacilli, the consumption of either the food product or food supplements in different doses does not affect its claimed oxidative stress-lowering effect in the blood of consumers.

Keywords: probiotic; *Lactobacillus* spp.; health effect

### Binding of Extracellular Matrix Molecules by Probiotic Bacteria

#### **Štyriak, I.**^1^, Nemcová, R.^2^
^1^Institute of Geotechnics SAS, Slovakia; ^2^University of Veterinary Medicine and Pharmacy in Košice, Slovakia

*Introduction:* The aim of this study was to investigate extracellular matrix (ECM) binding of selected bacterial isolates with probiotic features and compare them with commercially used probiotic bacteria with respect to their ECM binding. *Methods:* For this study, ECM molecules were immobilized in microtiter plates (mucin and fetuin) or on the surface of latex beads. *Results:* Porcine mucin was bound by all 10 probiotic strains tested with important inter-strain differences; seven strains were classified as strongly adherent and three as weakly adherent. On the other hand, fetuin binding was similar (A_570nm_ values between 0.200 and 0.289) for all 13 strains tested. Concerning the binding of ECM molecules tested by PAA, strongly positive (3) binding of bovine fibrinogen was expressed by strains from fermented food (*Lactobacillus rhamnosus* GG, *Lactobacillus casei* Shirota, and *Lactobacillus johnsonii* La1) and by *L. casei* L.c. and *Lactobacillus* sp. 2I3 as well as by *Lactobacillus plantarum* LP. The remaining bacteria showed positive (2) or weakly positive (1) binding of bovine fibrinogen. Strongly positive (3) binding of porcine fibronectin was observed only with two strains (*L. casei* Shirota and *L. plantarum* LP), however, all other strains also bound this molecule (PAA values of 1 or 2). Bovine lactoferrin was bound strongly (3) by *L. casei* L.c., *Lactobacillus* sp. 2I3, and *L. plantarum* LP. Human lactobacilli expressed only weak (1) binding of this molecule. The binding of transferrins was only weak (1) or in two cases slightly better (2); however, many strains did not bind transferrins, especially bovine apo-transferrin. Some animal strains (at least *L. casei* L.c. and *Lactobacillus* sp. 2I3) are comparable with commercially used strains with respect to their ECM binding ability. Since this feature is, from our point of view, very important for probiotic bacteria, these strains should be considered for wider use in fermented feed (or probiotic preparations) for animals. Ten bacteriocinogenic and non-bacteriocinogenic enterococci, isolates from chicken and rabbits, were examined for their binding of collagen, fibronectin, albumin, and vitronectin in tubes containing Nutrient Broth No. 2. Individual strains expressed binding of selected glycoproteins to various degrees. *Discussion:* This study is important because the fact that bacteriocinogenic bacteria can bind some proteins suggests that many useful bacteria could also be used as probiotic strains if they have useful properties for organisms. Three gut lactobacilli from piglets (*L. plantarum* L 5, *Lactobacillus paracasei* L 81, and *Lactobacillus fermentum* L 670) and *L casei* subsp. *pseudoplantarum* L.c. from a calf were examined by microtiter plate binding assay for their lectin-like binding activity after their cultivation on Rogosa agar and in MRS broth.

Keywords: extracellular matrix; binding; probiotics; lactobacilli; mucin

### Probiotics for Preventing Preterm Labor, and Changes in Serum Levels of IL-6, C-Reactive Protein, and Ferritin

#### **Suchánek, P**., Urdzík, P., Matta, M., Fialkovičová, V., Dudič, R., Strojný, L., Bomba, A. Faculty of Medicine, Pavol Jozef Šafárik University in Košice, Slovakia

*Introduction:* High rates of preterm delivery (i.e. above 10–12%) are prevalent in developing and developed regions around the world; preterm birth is associated with nearly 80% of fetal, neonatal, and infant deaths. Evidence as a whole suggests that the sub-acute inflammatory process is associated with spontaneous preterm delivery. The study presented here tested an alternative to antibiotics, that is the so-called probiotics (a type of bacterial therapy), to avert threatened preterm delivery. Probiotics have been tested mainly for treating infectious, inflammatory, and allergic conditions occurring in the intestinal, genitourinary, and respiratory tracts. Many of the beneficial effects of probiotics, except for changes in the microbiota, are related to their immune-modulating effects (i.e. increases in both immunological and anti-inflammatory activity). The study presented here investigated perorally used probiotics to interrupt the infectious/inflammatory process that leads to preterm delivery. *Methods:* The study aimed to evaluate the efficacy of specially designed probiotics (*Lactobacillus rhamnosus* GG,^®^ 13.2 mg, *Bifidobacterium animalis* subsp. lactis, BB-12^®^ 42.0 mg) administered as two capsules a day, each capsule containing more than 2.7 million CFU, for 4 weeks to prevent premature birth. We conducted tests to determine whether probiotics are efficacious to decrease serum proinflamatory interleukin 6 (IL-6), C-reactive protein, and ferritin. Thirty symptomatic pregnant women (uterine contraction and/or progress vaginal score) with intact membranes were admitted to the study after the 28th but before the 34th week of pregnancy with a singleton pregnancy and without a previous history of preterm delivery. Other exclusion criteria were: multiple gestation, cervical incompetence (cerclage in current gestation), a fetus with major congenital malformations in the current gestation, insulin dependent diabetes mellitus, systemic arterial hypertension under medication, clinical suspicion of low urinary tract infection, chronic asthma requiring intermittent therapy, continuous or recent corticotherapy (up to 1 month), hemolytic perinatal disease, or systemic lupus erythematosus. Fifteen enrolled women had used probiotics. All women were admitted to hospital and received the following treatments: bed rest, magnesium sulfate, beta mimetics, antibiotics, vaginal disinfection, and antenatal corticosteroids. *Results:* After 4 weeks, statistically significant reduced serum levels of all three biomarkers were observed in the probiotics group. None of the women gave birth prematurely or experienced adverse effects. *Discussion:* Although the use of probiotics appears to treat vaginal infections in pregnancy, there are currently insufficient data from trials to demonstrate any impact of oral probiotics on preterm birth.

Keywords: premature labour; probiotics; interleukin-6; C-reactive protein; ferritin

### The Role of Microbiota in Immunity Development and Chronic Diseases: Gnotobiology as a Tool

#### **Tlaskalova-Hogenova, H.**^1^, Štěpánková, R.^1^, Kozáková, H.^1^, Hudcovic, T.^1^, Rossmann, P.^1^, Hrnčíř, T.^1^, Kverka, M.^1^, Zákostelská, Z.^1^, Klimešová, K.^1^, Schwarzer, M.^1^, Kokešová, A.^1^, Šrůtková, D.^1^, Kobayashi, K.^2^, van Eden, W.^3^
^1^Insitute of Microbiology, Academy of Sciences of the Czech Republic, Prague, Czech Republic; ^2^Dana Farber Cancer Institute, Harvard University, Boston, Massachusetts, USA; ^3^Faculty of Veterinary Medicine, Utrecht University, The Netherlands

Most body surfaces are inhabited by a vast number of microorganisms representing the so-called normal microflora, the microbiota. Metagenomic approaches are currently being used to decipher the genome of the microbiota (microbiome), and, in parallel, functional studies are being performed to analyze the effects of the microbiota on the host. Gnotobiological methods are an indispensable tool for studying the consequences of bacterial colonization. Balanced interaction between commensal bacteria and the host mucosal immune system is a prerequisite for intestinal homeostasis. Our previous studies have shown that gut microbiota play a crucial role not only in mucosal but also systemic immunity development (Tlaskalova-Hogenova et al. Ann NY Acad Sci 1983; 409: 96–113; Petnicki-Ocwieja et al. Proc Natl Acad Sci USA 2009; 106: 15813–18). Impaired function of the mucosal barrier and dysfunctional microbiota (dysbiosis) are associated with negative consequences, such as translocation of microbial components, endotoxemia, and hyperactivation of the immune system. By exerting pathological effects, microbiota could participate in the pathogenesis of various inflammatory and neoplastic diseases. Animal models of human diseases are helping to elucidate the etiology and pathogenetic mechanisms involved in disease development. An invaluable tool for these studies are experimental models of human diseases reared in germ-free conditions and colonized by defined bacterial mixtures or strains. Using this approach, we demonstrated direct involvement of microbiota components in chronic intestinal inflammation and the development of colonic neoplasia (Klimesova et al. Inflamm Bowel Dis 2013; 19: 1266–77). Interestingly, the development of atherosclerosis in APO-E deficient mice fed a standard low cholesterol diet was accelerated in APO-E deficient mice reared under germ-free conditions (Stepankova et al. J Atheroscler Thromb 2010; 17: 796–804). We also addressed the question of whether the intestinal microbiota affect the induction of mucosal tolerance against the birch pollen allergen. Mice reared in germ-free and conventional conditions did not differ in their ability to induce oral tolerance. Colonization of germ-free mice at birth with probiotic recombinant *Lactobacillus plantarum* producing a clinically relevant allergen (Bet v 1) reduces experimental allergy and might become an effective strategy for early intervention against the onset of allergic diseases (Schwarzer et al. Allergy 2011; 66: 368–75). We found that some orally applied bacterial components from commensal or probiotic bacteria are able to prevent and treat experimentally induced intestinal inflammation (model of human inflammatory bowel disease) (Kverka et al. Clin Exp Immunol 2011; 163: 250–9). The identification of microbiota components and elucidation of the molecular mechanisms of their action when inducing pathological changes or exerting beneficial, disease protective activity could thus help to influence microbiota composition and/or immunoregulatory mechanisms involved in disease pathogenesis.

**Acknowledgments**

The work was supported by grants P304/11/1252 and 303/12/0535 (Czech Science Foundation) and NT/1348 (Grant Agency of the Ministry of Health of the Czech Republic).

Keywords: gut microbiota; chronic diseases; gnotobiology

### Different Levels of Energy Extraction in the Form of Short-Chain Fatty Acids from Fibers by Lean and Obese Microbiota: Implications for Obesity?

#### **Venema, K.**^1,4^, Aguirre, M.^1,2,4^, Bussolo de Souza, C.^1,3^
^1^TNO, The Netherlands; ^2^Maastricht University, The Netherlands; ^3^University of Groningen, The Netherlands; ^4^TI Food and Nutrition, The Netherlands

*Introduction:* The gut microbiota has been implicated to play a role in obesity, although the exact mechanism is not yet clear. It has become clear, however, that more than the ratio of Firmicutes versus Bacteroidetes, or the capacity the extract energy in the form of short-chain fatty acids (SCFA) from fermentable substrates is involved. *Aim:* Using TNO's validated *in vitro* model of the colon (TIM-2), we set out to study differences in energy extraction between the microbiota originating from lean volunteers and the microbiota from obese volunteers. *Materials and Methods:* Lean participants had an average body mass index (BMI) of 23.5±1.3 kg/m^2^, and obese participants a BMI of 33.5±2.6 kg/m^2^. Several different fiber substrates were tested and compared in the *in vitro* model. These included: lactulose, inulin, apple pectin, sugar beet pectin, galacto-oligosaccharides, and cassava starch. We analyzed microbiota composition using next generation sequencing. The production of SCFA and lactate as well as protein fermentation metabolites (ammonia, branched-chain fatty acids) were measured to determine microbiota activity. *Results and discussion:* In terms of prebiotic activity, inulin, lactulose, and galacto-oligosaccharides increased the bifidobacterial population in both microbiotas, but to a different degree. Interestingly, cassava starch also stimulated bifidobacteria in the lean microbiota to an approximately 10-fold greater extent than inulin. The growth of *Catenibacterium* spp., *Dorea* spp., *Clostridium* cluster *XIVb* spp., and *Faecalibacterium* was mostly affected by the pectins. When the amount of energy extracted in the form of SCFA was calculated, it was striking that the obese microbiota did not necessarily extract more energy from the different substrates. We hypothesize that the different microbiotas differ in their capacity to ferment the different substrates, due to differences in composition and therefore fiber degradation machinery. These differences in fiber degradation machinery lead to changes in energy extraction which then become dependant on the substrates provided to the microbiota, for example, pectin may not be a substrate ingested much by obese volunteers and it seems from our results that the microbiota from these obese individuals have a lower capacity to ferment this substrate, leading to lower energy extraction compared to a lean microbiota. *Conclusions:* The microbiotas from lean and obese volunteers vary in their composition and thus in their capacity to break down different fiber substrates. This difference in fiber degradation capacity defines their efficiency regarding energy extraction from the different substrates. There does not seem to be a general rule that an obese microbiota extracts more energy from a given substrate than a lean microbiota. *Future perspectives:* We are currently testing the effects of samples from TIM-2 on signaling of the host, with respect to immunomodulation, satiety hormone production, etc. It will likely be a combination of microbiota composition, microbiota activity, and host-signaling will determine the outcome of the influence of the microbiota on obesity. A particular fiber may have opposite effects in lean versus obese individuals.

Keywords: obesity; microbiota; in vitro; energy extraction

### Development of Gut Microbiota of Egg Layers and Changes Induced by Antibiotic Therapy

#### **Videnska, P.**^1^, Faldynová, M.^1^, Sisak, F.^1^, Havlickova, H.^1^, Sedlar, K.^2^, Rychlik, I.^1^
^1^Veterinary Research Institute, Brno, Czech Republic; ^2^Brno University of Technology, Brno, Czech Republic

*Introduction:* The gut colonization of chickens in commercial production facilities immediately after hatching is dependent only on environmental sources because of the total absence of hens as donors of healthy microbiota, and it is unclear to what extent the gut microbiota composition in egg layers is dynamic or stable. In this study we therefore characterized the development of the gut microbiota of laying hens during their life and changes induced by tetracycline therapy. *Methods:* The microbiota in the cecum of egg laying hens (Lohman Brown Light) was characterized from the first week of their life until week 60. In single-cycle therapy, we characterized the microbiota in the feces of 15-week-old layers prior and 2 days after antibiotic therapy with tetracycline. In repeated therapy, we characterized the gut microbiota of 46-week-old laying hens. These were subjected to 2 days of therapy which was repeated for an additional 2 days after 12 days of antibiotic withdrawal. Purified caecal or fecal DNA was used as a template in PCR amplifying V3/V4 variable regions of 16S rRNA genes. Amplification products were then sequenced using a GS Junior 454 sequencer according to the manufacturer's instructions (Roche) and data were analyzed using QIIME software. *Results:* Representatives of 10 different phyla were recorded at least once in the caecal microbiota of hens during their whole life. However, representatives of three phyla, Proteobacteria, Firmicutes, and Bacteroidetes, formed the vast majority of the microbiota in all age categories. We observed four developmental stages. The first stage lasted for 1 week and was characteristic by the increased presence of representatives of the phylum Proteobacteria. The second stage of microbiota development lasted for 3 weeks, and was characteristic by absolute dominance of the representatives of families *Lachnospiraceae* and *Ruminococcaceae* (phylum Firmicutes). The third stage of microbiota development lasted until 4 months of life. During this stage, a gradual succession of representatives of Firmicutes and their replacement with representatives of Bacteroidetes was observed. The fourth stage was characteristic for hens older than 4 months. The microbiota in this age category was formed by representatives of Firmicutes and Bacteroidetes and representatives of each of the phyla formed approximately half of the total microbiome. The complexity of the gut microbiota decreased by up to 94% after antibiotic administration and representatives of *Enterobacteriaceae* and *Enterococcaceae* were repeatedly resistant to both antibiotics. *Conclusions:* We observed four stages of development of microbiota during a whole life of laying hens. The changes in microbiota composition induced by the antibiotic therapy were rapid and quite dramatic and only representatives of the genera *Enterococcus* and *Escherichia* increased in response to the antibiotic therapy. Interestingly, restoration of microbiota complexity after therapy withdrawal was nearly as rapid as the changes immediately after therapy.

Keywords: gut; microbiota; chicken; antibiotics

### DNA-Microarray for Genotyping Antibiotic Resistance Determinants in *Acinetobacter baumannii* Clinical Isolates

#### **Weile, J.**^1^, Dally, S.^1^, Lemuth, K.^2^, Rupp, S.^3^, Knabbe, C.^1^
^1^Heart and Diabetes Center NRW, Bad Oeynhausen, Germany; ^2^Bosch GmbH, Stuttgart, Germany; ^3^Fraunhofer IGB, Stuttgart, Germany

*Introduction:* The development of antibiotics at the beginning of the 20th century led to a dramatic decrease in deaths from infectious diseases. However, bacteria have the ability to develop resistance through mutation or gene acquisition, and to spread this resistance via horizontal gene transfer to other species. After the introduction of a new antibiotic agent, the development of a corresponding resistance determinant is merely a matter of time. About a century of antibiotic usage has led to a huge repertoire of bacterial resistance determinants. This diversity makes the detection and identification of the causative gene in a resistant isolate challenging. *Acinetobacter baumannii* is an opportunistic pathogen which can cause severe infections, particularly pneumonia, sepsis, urinary tract infections, and wound infections. Although *A. baumannii* is not widespread in the community, it is one of the most frequently occurring pathogens causing outbreaks on intensive care units, and is associated with high mortality and morbidity rates. Microarray technology offers a technique capable of detecting a huge number of different genes simultaneously in a short period of time. *Methods:* In order to improve the diagnosis of resistance determinants in terms of lead time and accuracy, we developed a microarray capable of simultaneously detecting 91 target sequences associated with antibiotic resistance in *A. baumannii*. The developed assay consisted of the following steps: nucleic acid extraction, multiplex-PCR amplification of target sequences, microarray hybridization, and data read-out. The array was validated in a blinded, prospective study with 60 multi-drug-resistant strains of *A. baumannii*, and its results were compared with the phenotype determined by the automated susceptibility testing system VITEK2. Considered antibiotics were piperacillin/tazobactam, ceftazidime, imipenem, meropenem, trimethoprim/sulfamethoxazole, amikacin, gentamicin, tobramycin, ciprofloxacin, and tigecycline. *Results:* The average positive predictive value, negative predictive value, sensitivity, and specificity were 98%, 98%, 99%, and 94%, respectively. Regarding carbapenemase production, the array results were compared with singleplex-PCR results provided by the German National Reference Center for Gram-Negative Pathogens. The accordance between them was 100%. Positive probes were, with an average signal of about 15,000 counts, clearly distinguishable from negative probes, which showed an average signal of 20 counts. Analysis processing time from bacterial culture to result was about 4 h. *Conclusions:* These data demonstrate that the developed array is a reliable tool capable of detecting manifold causes of resistance in parallel. Consequently, the array may help to provide fast results in order to initiate adequate therapy against infection in critically ill patients, as well as data acquisition for epidemiological surveillance.

Keywords: *Acinetobacter baumannii*; microarray; antibiotic resistance; genotyping; epidemiologic surveillance

### The Effect of Ciprofloxacin on the Composition of the Microflora and the Emergence of Antibiotic Resistance in Volunteers

#### **Weintraub, A**., Rashid, M.-U., Sorensson, M., Wahlund, E., Nord, C.E. Karolinska Institutet, Sweden

*Objectives:* The purpose of this study is to assess the effect of ciprofloxacin (500 mg bd for 10 days) on the skin, nasal, saliva, and intestinal microflora of healthy humans for a period of 1 year. In addition, the consequences of the emergence and persistence of ciprofloxacin resistant bacteria in the normal microflora were studied. *Methods:* Twenty healthy subjects (10 males and 10 females) were randomly assigned in the study. Ten volunteers were given ciprofloxacin (500 mg, bid) for 10 days while the remaining 10 volunteers were given a placebo. Skin, nasal, saliva, and fecal samples were collected for analysis on six occasions: immediately before administration, day 11, 1 month, 2 months, 4 months, and 12 months after dosing. The aerobic non-selective and selective agar plates were incubated for 24 h at 37°C and anaerobic non-selective and selective agar plates for 2–7 days at 37°C in anaerobic jars. After incubation, different colony types were counted, isolated in pure culture, and identified. *Results:* In the skin microflora, there were no effects on *Propionibacterium acnes* or other bacteria and only a minor effect on coagulase-negative staphylococci (CoNS) that normalized after 4 months. In the nasal microflora, there were minor effects on CoNS and *P. acnes* that normalized after 2 months. In the saliva microflora, there were minor effects on alpha-hemolytic streptococci and Fusobacteria that normalized after 4–12 months. There were no effects on *Candida albicans*, Lactobacilli, Leptotrichia, Prevotella, *Streptococcus salivarius*, or Veillonella. In the intestinal microflora, there were no effects on the *Enterobacteriaceae* or *Candida albicans*. Initially, there was no effect on the enterococci, but after 4 months there was an increase that normalized after 1 year. There were major effects on *Escherichia coli* and on bifidobacteria that normalized after 2 months. There were minor effects on Lactobacilli and Bacteroides. Detectable concentrations of ciprofloxacin were only found in the fecal samples in all the volunteers receiving the antibiotic and only on day 11, that is, directly after the treatment. *Conclusions:* The interval for normal microflora to be normalized after ciprofloxacin treatment is different for different bacterial species and body sites. It takes 2–12 months to normalize the human microflora after treatment. In the present study, no *Clostridium difficile* colonization or *C. difficile* infections were reported. The research leading to these results received funding from the European Union Seventh Framework programme (FP7/2007-2013) under grant agreement no. 241446.

Keywords: ciprofloxacin; normal microflora; long-term effect

### The Effect of Clindamycin on the Composition of the Microflora and the Emergence of Antibiotic Resistance in Volunteers

#### **Weintraub, A**., Rashid, M.-U., Sorensson, M., Wahlund, E., Nord, C.E. Karolinska Institutet, Sweden

*Objectives:* The purpose of this study is to assess the effect of clindamycin (150 mg qid for 10 days) on the skin, nasal, saliva, and intestinal microflora of healthy humans for a period of 1 year. In addition, the consequences of the emergence and persistence of clindamycin resistant bacteria in the normal microflora were studied. *Methods:* After undergoing screening, 20 healthy subjects (10 males and 10 females) were randomly assigned in the study. Ten volunteers were given clindamycin (150 mg qid) for 10 days, while the remaining 10 volunteers were given a placebo. Skin, nasal, saliva, and fecal samples were collected for analysis on six occasions: immediately before administration, day 11, 1 month, 2 months, 4 months, and 12 months after dosing. The aerobic non-selective and selective agar plates were incubated for 24 h at 37°C and anaerobic non-selective and selective agar plates for 2–7 days at 37°C in anaerobic jars. After incubation, different colony types were counted, isolated in pure culture, and identified. *Results:* In the skin microflora, there was no effect on *Propionibacterium acnes* and only minor effects on Coagulase-negative staphylococci (CoNS) and other bacteria. The levels normalized after 2–4 months. In the nasal microflora, there were minor effects on CoNS, *P. acnes*, and other bacteria that normalized after 4–12 months. In the saliva microflora, there were minor effects on *Streptococcus salivarius*, Fusobacteria, Leptotrichia, and Prevotella that normalized after 1–4 months. There were no effects on *Candida albicans*, Lactobacilli, or Veillonella. In the intestinal microflora, there were no effects on the *Enterobacteriaceae*, *Escherichia coli*, or *Candida albicans*. There were minor effects on enterococci, bifidobacteria, lactobacilli, and Bacteroides that normalized after 2–12 months. No *Clostridium difficile* was detected in any of the samples. Clindamycin was only found in the stool samples from volunteers receiving the antibiotic and only directly after treatment, that is, on day 11. *Conclusions:* The interval before normal microflora are normalized after clindamycin treatment is different for different bacterial species at different body sites. It takes 1–12 months to normalize the human microflora after clindamycin treatment. In the present study, no *C. difficile* colonization or *C. difficile* infections were reported. The research leading to these results received funding from the European Union Seventh Framework programme (FP7/2007-2013) under grant agreement no. 241446.

Keywords: clindamycin; normal microflora; long-term effect

### An Overview of the ANTIRESDEV Project

#### Wilson, M. University College London, UK

ANTIRESDEV is a €5.4M EU funded research project in the ‘Health’ theme of the Seventh RTD Framework Programme. The objectives of the project are to use a multidisciplinary approach to study the impact of different antibiotics in selecting resistance among pathogenic and commensal members of the indigenous microbiota of humans and to determine their effects on the composition of indigenous microbial communities using culture-dependent and culture-independent techniques. The project has involved:

 (1) carrying out longitudinal intervention studies on healthy volunteers using antibiotics with different modes of action, antimicrobial spectra, and pharmacokinetic properties, namely clindamycin, ciprofloxacin, amoxicillin, and minocycline

(2) identifying the antibiotic-resistant bacteria that arose from their use

(3) determining the dynamics of the emergence and persistence of the antibiotic-resistant strains isolated

(4) modeling the ecological impact of antibiotic administration on the indigenous microbiota

(5) developing and using state-of-the-art microarrays to identify the genetic basis of resistance in resistant isolates

(6) identifying the genetic elements mediating the transmission of resistance

(7) characterizing the likely mode of transmission and mobility of selected elements

(8) determining the biological cost to the organisms of developing resistance to one or more antibiotics since this impinges on the dynamics and transmission of antibiotic resistance throughout the population

(9) using a culture-independent approach (454 pyro-sequencing) to determine the composition of the oral and fecal microbiotas (including not-yet-cultivated members)

(10) identifying the full complement of antibiotic resistance determinants (the ‘resistome’) in these communities and the effect of antibiotic administration on them.

Keywords: antibiotic resistance

### Thermostable Probiotics: A Promising Tool for Restoring Vaginal Health

#### **Yamamoto, H.**^1^, Bronshtein, V.^2^, Rohan, L.C.^3^, Onderdonk, A.B.^1^, Fichorova, R.N.^1^
^1^Brigham and Women's Hospital and Harvard Medical School, Boston, MA, USA; ^2^Universal Stabilization Technologies, Inc, San Diego, CA, USA; ^3^Magee Women's Research Institute, University of Pittsburgh, Pittsburgh, PA, USA

*Introduction:* Bacterial vaginosis (BV) is a common mucosal microbiome disturbance especially affecting women of reproductive age. BV is associated with an increased risk of reproductive outcome complications and acquisition of sexually transmitted infections including HIV. Probiotic products have been sought to repopulate beneficial vaginal bacterial communities in women with BV and to serve as natural anti-HIV agents. However, a barrier to their clinical efficacy and more ubiquitous use is the limited stability of bacteria at ambient temperatures in traditional formulations offered over the counter. Here we describe a novel thermostable vaginal probiotic with beneficial immune properties. *Methods: Lactobacillus jensenii* (Lj), *Lactobacillus rhamnosus* (Lgg), and *Lactobacillus crispatus* (Lc), Gram-positive bacteria used as indicators of vaginal health, were subjected to preservation by vaporization (PBV) technology and tested after exposure to room temperature for 3–12 months. The PBV bacteria were incorporated into a quick dissolve thin film formulation as a vehicle to deliver the live probiotic to the female genital tract. We hypothesize the PBV bacteria in this formulation would maintain their functional properties allowing viable lactobacilli to actively colonize the female reproductive tract and preserve the mucosal innate-immunity balance. We compared PBV bacteria, the film formulation, and the same bacteria preserved by traditional freezing technology. All bacteria were co-cultured with human cervical and vaginal epithelial cells for 4, 24, and 48 h. Colony forming units (CFU) on Brucella-based agar were used to examine the recovery of viable bacteria. Tissue toxicity was measured by MTT and LDH assays with Triton X as positive toxicity control. Innate immune mediators were measure from cell culture supernatants by ELISA or electrochemiluminescence assay using MALP-2 as a pro-inflammatory control and staurosporine as a control for apoptosis. *Results:* There was no significant variation in CFU between PBV bacteria kept at ambient temperature and the original bacterial strains kept frozen. All bacteria were 100% recoverable from the dissolved film formulation, suggesting a lack of bacterial damage in both the PBV and the film manufacturing processes. The dissolved film formulations colonized the epithelial cells at consistent rates causing no toxicity. Both the film formulation and the PBV bacteria alone caused no unwanted immunoinflammatory responses measured by pro-inflammatory (NF-κB, IL-1β, IL-6, and IL-8) and anti-inflammatory (IL-1RA, SLPI, and Elafin) biomarkers. *Discussion:* Lj, Lgg, and Lc when combined with the preservation by vaporization technology and a quick-dissolved films formulation maintain high viability, reliable colonization properties, and desired intact mucosal immune barrier.

Keywords: probiotics; vaginal film; cytokines; inflammation

### The Effects of Different Antibiotics on Fecal Microbiome Profiles by 16S rRNA Gene Amplicon Sequencing

#### Zaura, E. Consortium ANTIRESDEV ACTA, The Netherlands

*Introduction:* The human body is colonized by a vast number of microbes, collectively called the human microbiome. Here we aimed to determine the ecological impact of the use of antibiotics on the diversity and relative abundance of phylotypes of the indigenous fecal microbiota. *Methods:* Fecal samples were collected from healthy individuals during four clinical studies before (baseline), immediately after (week 1) administration of the antibiotic (minocycline, amoxicillin, ciprofloxacin, and clindamycin) or placebo and 1, 2, 4, and 12 months later. The DNA was extracted and quantified, and barcoded amplicon libraries of the small subunit ribosomal RNA gene hypervariable region V5–V7 were generated for each of the individual samples, pooled, and sequenced by means of the Genome Sequencer FLX Titanium system. The sequencing data were processed using QIIME version 1.5.0. Quality filtered reads were denoised (Denoiser version 1.3.0) and chimeric reads identified (UCHIME version 4.2.40). The cleaned reads were clustered into operational taxonomic units (OTUs) at 97% similarity using the UCLUST Optimal clustering algorithm and the taxonomy assigned using the RDP classifier. Phylogenetic and diversity analyses were performed on randomly subsampled datasets. *Results:* The microbiomes from the placebo groups showed stable species richness and diversity throughout the year of the study. The samples obtained from the same individual showed high phylogenetic relatedness. The baseline and week 1 samples from the same individual in the placebo groups were highly similar. Some fluctuation in microbial composition was observed between more distant timepoints. Fecal samples from the minocycline group showed a significant decrease in species richness up to day 30. A shift in composition was observed right after exposure to minocycline but had recovered by day 30. Exposure to amoxicillin had no significant effect on the diversity of the fecal microbiome. Shifts in microbial proportions (quantitative changes) were observed right after exposure to the antibiotic and showed a recovery by day 30. Exposure to ciprofloxacin had a strong and prolonged effect on the diversity of the fecal microbiome: even 1 year after antibiotic exposure, species richness and the diversity index were significantly lower than at baseline. Pronounced qualitative and quantitative compositional shifts were observed by day 30 and a recovery in composition by day 120. Exposure to clindamycin had a strong effect on the diversity of the fecal microbiome that was still significant on day 120. A qualitative microbial shift in composition was observed until day 120, with the most pronounced shift present on day 30. The microbiomes exposed to clindamycin showed recovery in diversity and composition only after 1 year. *Discussion:* All antibiotics had an impact on the fecal microbiome. Depending on the antibiotic, the impact varied from less than 1 month to more than 1 year. Several antibiotics reduced the proportion of bacteria known to produce butyrate (e.g. Faecalibacterium, Roseburia, Lachnospiraceae, Ruminococcaceae). Butyrate is an important substrate for the colonocytes and is important in maintaining gastrointestinal health. Future studies should address the possible effects of antibiotics on butyrate production and colonocyte physiology.

Keywords: microbiome; faeces; antibiotics

